# Does the Genomic Landscape of Species Divergence in *Phaseolus* Beans Coerce Parallel Signatures of Adaptation and Domestication?

**DOI:** 10.3389/fpls.2018.01816

**Published:** 2018-12-19

**Authors:** Andrés J. Cortés, Paola Skeen, Matthew W. Blair, María I. Chacón-Sánchez

**Affiliations:** ^1^Corporación Colombiana de Investigación Agropecuaria (Agrosavia) – Centro de Investigación La Selva, Rionegro, Colombia; ^2^Universidad Nacional de Colombia – Sede Medellín, Facultad de Ciencias Agrarias – Departamento de Ciencias Forestales, Medellín, Colombia; ^3^Universidad Nacional de Colombia – Bogotá, Facultad de Ciencias Agrarias – Departamento de Agronomía, Bogotá, Colombia; ^4^Department of Plant Sciences, University of California, Davis, Davis, CA, United States; ^5^Department of Agricultural and Environmental Science, Tennessee State University, Nashville, TN, United States

**Keywords:** genomic islands of speciation, genomic signatures of selection, domestication syndrome, convergent evolution, gene flow, genomics constrains, GBS-derived SNP markers

## Abstract

Exploring the genomic architecture of species and populations divergence aids understanding how lineages evolve and adapt, and ultimately can show the repeatability of evolutionary processes. Yet, the genomic signatures associated with divergence are still relatively unexplored, leading to a knowledge gap on whether species divergence ultimately differs in its genetic architecture from divergence at other spatial scales (i.e., populations, ecotypes). Our goal in this research was to determine whether genomic islands of speciation are more prone to harbor within-species differentiation due to genomic features, suppressed recombination, smaller effective population size or increased drift, across repeated hierarchically nested levels of divergence. We used two species of *Phaseolus* beans with strong genepool and population sub-structure produced by multiple independent domestications each especially in Andean and Mesoamerican / Middle American geographies. We genotyped 22,531 GBS-derived SNP markers in 209 individuals of wild and cultivated *Phaseolus vulgaris* and *Phaseolus lunatus*. We identified six regions for species-associated divergence. Out of these divergence peaks, 21% were recovered in the four within-species between-genepool comparisons and in the five within-genepool wild-cultivated comparisons (some of the latter did retrieve genuine signatures of the well described multiple domestication syndromes). However, genomic regions with overall high relative differentiation (measured by F_ST_) coincided with regions of low SNP density and regions of elevated delta divergence between-genepools (Δ_Div_), independent of the scale of divergence. The divergence in chromosome Pv10 further coincided with a between-species pericentric inversion. These convergences suggest that shared variants are being recurrently fixed at replicated regions of the genome, and in a similar manner across different hierarchically nested levels of divergence, likely as result of genomic features that make certain regions more prone to accumulate islands of speciation and within-species divergence. In summary, neighboring signatures of speciation, adaptation and domestication in *Phaseolus* beans are influenced by ubiquitous genomic constrains, which may continue to fortuitously shape genomic differentiation at various others scales of divergence.

## Introduction

Exploring the genomic architecture of species’ and populations’ divergence helps understanding how lineages evolve and adapt, and ultimately how repeatable evolutionary processes are (Gould, 1990; Lobkovsky and Koonin, 2012; Tenaillon et al., 2012). Recent sequencing studies have demonstrated that the genomic consequences of divergence are mixed (Ellegren and Wolf, 2017; Ravinet et al., 2017) and even misleading (Pennisi, 2014). Therefore, a knowledge gap on whether species divergence differs in its genetic architecture from divergence at other spatial scales (i.e., populations, ecotypes) still remains. Addressing this knowledge breach is a much-needed step to fully comprehend the relative contribution of variable gene flow and drift, divergent natural and human selection, and genome-wide heterogeneous recombination and effective population size in shaping the genomic landscape of divergence across and within species.

Genomic signatures associated with species, genepools and ecotypes’ divergence can result from reduced gene flow due to interspecific cross-incompatibility, but also random genetic drift and selection (Nei, 2010). The origin of outlier variants from novel or standing genetic variation leads to distinctively different patterns of genomic divergence (Hermisson and Pennings, 2005; Barrett and Schluter, 2008; Pritchard et al., 2010). One approach that can help to distinguish these underlying causes of divergence is carrying out a replicated sampling of contrasting populations (Roesti et al., 2014; Lotterhos and Whitlock, 2015). If genetic drift rather than selection is responsible for the divergence, it is unlikely that signals of differentiation reappear consistently across replicates (Lotterhos and Whitlock, 2015). On the other hand, if selection acted on the same genetic variants at the replicated contrasting pairs, genomic regions with comparatively high divergence between individuals from contrasting populations should be identical at each of the replicated populations and those are likely to contain genes involved in adaptive divergence (Nielsen, 2005). Parallel selection on shared genetic variation, like in the well-described *Atp1a1, EDA* and *Pitx1* genes in sticklebacks (Jones et al., 2012a,b), should therefore lead to low divergence within populations and across replicates, in the exact genomic regions where equivalent variants are selected at each contrasting population (Roesti et al., 2014). In other words, divergent regions would co-localize with regions of reduced divergence in within-population comparisons if those regions differentiated as a result of parallel divergence from shared variation rather than due to novel variation evolving at each site (Roesti et al., 2014; Irwin et al., 2016). Discerning among gene flow, genetic drift and selection as the cause of parallel genomic divergence is possible as long as there is some degree of replication considered in the sampling of contrasting populations.

The genomic landscape of divergence can also be influenced by differences in ancestral variation and recombination in the genome (Strasburg et al., 2011; Wolf and Ellegren, 2016). Lineage sorting may be enhanced relative to background levels by a reduction in the effective population size (N_e_) due to processes other than gene flow, like low recombination (Jones et al., 2012b; Zhou et al., 2014; Wolf and Ellegren, 2016), creating genomic islands that eventually expand by genetic hitchhiking (Ma et al., 2018). Since differentiation is further speeded up in low-recombining regions because of linked selection (Ellegren and Galtier, 2016), the imprint caused by genomic features on the differentiation landscape should be ubiquitous across different levels of divergence. Comparing relative (F_ST_) differentiation (Nachman and Payseur, 2012; Cruickshank and Hahn, 2014; Irwin et al., 2016) across hierarchical nested scales of divergence (Pereira et al., 2016), and coupling this with estimates of the recombination rate (Maynard Smith and Haigh, 1974; Nielsen, 2005; Storz, 2005), can therefore allow for further inferences on the processes giving rise to parallel divergence patterns, such as genomic constrains (Hermisson and Pennings, 2005; Barrett and Schluter, 2008; Pritchard et al., 2010). Therefore, besides a replicated sampling of contrasting populations, a hierarchical nested sampling across various scales of divergence is advisable in order to examine whether genomic islands of divergence may display differentiation due to suppressed recombination, smaller effective population size and increased drift.

*Phaseolus* beans, with their striking genepool structure and multiple domestications, constitute an excellent model system (Broughton et al., 2003; Bitocchi et al., 2017) to test these approaches and to explore to what extent genomic features, besides reduced gene flow and divergent selection, may lead to genomic divergence between (i.e., speciation islands) and within species (i.e., during the natural colonization of new habitats as well as part of the domestication syndromes, which is the suite of phenotypic and genetic changes arising during domestication that distinguish crops from their wild ancestors). Common and Lima beans are the only bean species with multiple domestications among the five domesticated species of *Phaseolus* (Bitocchi et al., 2017). Wild common bean (*P. vulgaris* L.) diverged from its sister species in the tropical Andes (Rendon-Anaya et al., 2017) and colonized South and Central America, originating what nowadays are known as the Andean and Mesoamerican genepools. Independent domestication events for each genepool gave rise to the Andean and Mesoamerican cultivars, although the exact location is still under debate, with a strong role of wild to cultivated introgression likely in the development of cultivar races (Gepts and Debouck, 1991; Kwak and Gepts, 2009; Schmutz et al., 2014). Similarly, wild Lima bean (*P. lunatus* L.) diverged from other *Phaseolus* species in the Andes, after which natural spread also led to a strong genepool structure, with the development of two Andean and two Mesoamerican genepools. Furthermore, independent domestications happened at least in two of these genepools, creating a very diverse range of phenotypes/genotypes for wild and cultivated lima beans that is still under exploration and only recently been analyzed (Chacon-Sanchez and Martinez-Castillo, 2017). To date, genotyping by Sequencing – GBS (Elshire et al., 2011) is among the preferable marker systems for *de novo* common bean SNP detection given its versatility through a wide range of applications (see Cortés and Blair, 2018a, and references therein, and compare with Cortés et al., 2011; Galeano et al., 2012; Kelleher et al., 2012; Blair et al., 2013). This technique has recently been implemented in Lima bean as well (Chacon-Sanchez and Martinez-Castillo, 2017). Therefore, common and Lima beans not only offer an exceptional arrangement of replicated hierarchical-nested scales of divergence to test a wide spectrum of debatable evolutionary hypotheses, but also possess the necessary genetic resources to accurately quantify genome-wide patterns of differentiation.

In this study we took advantage of the recurrent phylogeographic splits and nested domestication events of common and Lima beans to examine whether genomic islands of speciation in *Phaseolus* species are more prone to harbor within-species divergence due to reduced recombination and increased drift. To accomplish this goal, we asked (1) are between-species F_ST_ outliers recovered in within-species comparisons, (2) is there any parallelism in the within-species divergence F_ST_ profiles, and (3) does divergence across repeated and hierarchically nested scales of divergence correlate with intrinsic genomic features (i.e., low-recombining regions such as centromeres and chromosomal rearrangements)? We predicted that if there were some parallelisms in the genetic adaptations to the Mesoamerican and Andean environments or in the genetic consequences of the domestication syndromes, then there would be matching signals of differentiation in the within-species between-genepools divergence F_ST_ profiles, or in the within-genepool wild-cultivated divergence F_ST_ profiles, respectively. These patterns of repeatability would not be observed if between-genepools and wild-cultivated divergence outliers were due to genetic drift (Lotterhos and Whitlock, 2015), if selection pressures were different (Ravinet et al., 2017), or if equivalent selective forces did not act on the same shared variation (Roesti et al., 2014; Ravinet et al., 2016). Yet, genomic constrains, rather than true signals of convergent adaptation and domestication, could still be the reason for these parallelisms. If genomic features were indeed constraining divergence, then genomic islands of differentiation would coincide with low-diversity and low-recombining regions, as well as with chromosomal rearrangements, regardless the nature and the scale of divergence.

## Materials and Methods

### Plant Material

A total of 209 accessions from *Phaseolus vulgaris* and *Phaseolus lunatus* were used in this study (Supplementary Table S1). All the genotypes were provided by the Genetic Resources Unit at the International Center for Tropical Agriculture (CIAT) and are preserved under the treaty for genetic resources from the Food and Agriculture Organization (FAO). These accessions are representative samples of the core collections for wild (Tohme et al., 1996) and cultivated common (Díaz and Blair, 2006; Blair et al., 2007) bean; as well as a diverse sampling of Lima beans (Motta-Aldana et al., 2010; Serrano-Serrano et al., 2010; Martínez-Castillo et al., 2014), for which there is not yet a core collection. All genepools and races/subpopulations for the wild and cultivated accessions of the two species have already been uncovered by the marker studies of Blair et al. (2012) and Chacon-Sanchez and Martinez-Castillo (2017). A total of 79 materials from *P. vulgaris* comprised 52 Mesoamerican (22 wild and 30 cultivated) and 27 Andean (9 wild and 18 cultivated) accessions; whereas a total of 130 materials from *P. lunatus* comprised 18 Andean I (8 wild and 10 cultivated), 9 Andean II (all wild), 59 Mesoamerican I (16 wild and 43 cultivated) and 44 Mesoamerican II (33 wild and 11 cultivated) accessions. This sampling spanned all genepools and wild and cultivated populations within species across a replicated hierarchical-nested framework of divergence (Supplementary Figure S1).

### Sample Collection, DNA Extraction and Genotyping-by-Sequencing

Leaf tissue weighing approximately 20 mg was harvested at 40 days after plant germination and was immediately dried in Silica Gel (Sigma-Aldrich, Germany). Genomic DNA was extracted using the QIAGEN DNeasy Plant Mini Kit (QIAGEN, Germany), following the manufacturer’s instructions, and quantified using a Qubit^®^ dsDNA HS Fluorometer (Life Technologies, Stockholm, Sweden). Three 96-plex genotyping-by-sequencing (GBS) assays were made according to Elshire et al. (2011) for the 209 accessions, with one accession per assay chosen at random as duplicate. Library preparation with *ApeKI* digestions and paired-end sequencing were performed at the Biotechnology Resource Center of the Institute for Genomic Diversity (Cornell University, United States) and at the Australian Genome Research Facility (Melbourne, VIC, Australia). Genotyping and SNP calling were analyzed with the software NGSEP (Duitama et al., 2014). Sequence tags were aligned to the *P. vulgaris* v.2.1 reference genome (Schmutz et al., 2014), which is well-annotated and in high synteny with the *P. lunatus* genome (Bonifácio et al., 2012), using the BWA method (Li and Durbin, 2007). Trait Analysis by association, Evolution and Linkage (TASSEL) software (Glaubitz et al., 2014b) was used for filtering.

### Overall Patterns of Population Structure

We explored per-species genepool and subpopulation structure in the 209 accessions of common and Lima beans using principal coordinates analysis (PCoA) implemented in TASSEL (Glaubitz et al., 2014a). Customized scatter plots were drawn using R v.3.3.1 (R Core Team) and were colored according to the domestication status (wild or cultivated) and the genepool/race/subpopulation identity, following Blair et al. (2009, 2012) and Chacon-Sanchez and Martinez-Castillo (2017). Based on this exploration, Andean wild accessions of common bean, from the Ecuador north-Peru subpopulation, and Andean II wild accessions of Lima bean were included as controls for population structure but excluded from the oncoming analyses because they aggregated into independent genetic clusters with low representation, as had already been reported by Blair et al. (2012) and Chacon-Sanchez and Martinez-Castillo (2017).

### Patterns of Genomic Divergence

Patterns of genomic divergence were explored at three different hierarchically nested levels, based on the overall trends of population structure revealed in the previous section. At the top level, species-associated divergence (*P. lunatus* vs. *P. vulgaris*) was used as a proxy to identify islands of speciation, following Nosil and Feder (2011). At the intermediate level, within-species between-genepools divergence was considered as a consequence of microevolution, that is population divergence and potentially different adaptations to the Mesoamerican and the Andean regions. This level contained four comparisons, one within *P. vulgaris* (Andean vs. Mesoamerican genepools) and three within *P. lunatus* (Andean I vs. Mesoamerican I, Andean I vs. Mesoamerican II and Mesoamerican I vs. Mesoamerican II genepools). At the bottom level, within-genepool wild-cultivated comparisons were indicative of the domestication syndrome. This level contained a total of five comparisons, two within *P. vulgaris* (wild vs. cultivated within the Andean and Mesoamerican genepools) and three within *P. lunatus* (wild vs. cultivated within the Andean I, Mesoamerican I and Mesoamerican II genepools). This set up led to a total of nine different comparisons of divergence, four of which are replicated within-species between-genepools, and five of which are replicated within-genepool wild-cultivated comparisons.

A sliding window analysis (window size = 1 × 10^7^ bps, step size = 500 kb) was used for contrasting between individuals at each of the ten comparisons. Window and step sizes had already been optimized in similar GBS-based genomic scans carried out in *Phaseolus* (Cortés and Blair, 2018a). We calculated relative differentiation computed as the fixation index – F_ST_ (Weir and Cockerham, 1984) and delta divergence – Δ_Div_ (Roesti et al., 2014). F_ST_ values were averaged across replicated comparisons within each level but were also kept independent for comparative purposes. Confidence intervals around all per-comparison genome-wide average F_ST_ estimates were computed using the R *quantile* function at an α value of 0.05. F_ST_ outliers were also identified at an α of 0.05 based on the overall F_ST_ distribution for each comparison. Calculations were done using Tassel v.5 (Bradbury et al., 2007) and customized R scripts. Results of all windowed analyses were plotted against window midpoints in millions of base pairs (Mb) also using R v.3.3.1 (R Core Team).

In order to make sense of the landscape of genomic divergence, we conducted the following comparisons:

(1) We analyzed whether the F_ST_ outliers between species coincided with high F_ST_ values at within-species comparisons. This pattern is expected if genomic islands of speciation are repeatedly more prone to harbor within-species divergence as a result of limited recombination (Wolf and Ellegren, 2016).
(2) We further assessed whether the within-species between-genepools divergence F_ST_ profiles were similar among the four available comparisons (Andean vs. Mesoamerican genepools of *P. vulgaris*, and Andean I vs. Mesoamerican I, Andean I vs. Mesoamerican II and Mesoamerican I vs. Mesoamerican II genepools of *P. lunatus*). This trend is expected if the same variants were selected as the result of similar selective pressures at the Mesoamerican and the Andean regions but not if divergence outliers were due to population divergence, that is genetic drift (Lotterhos and Whitlock, 2015).
(3) We also assessed whether the within-genepool wild-cultivated divergence F_ST_ profiles were similar among the five available comparisons (wild vs. cultivated *P. vulgaris* within the Andean and Mesoamerican genepools, and wild vs. cultivated *P. lunatus* within the Andean I, Mesoamerican I and Mesoamerican II genepools). This coincidence is expected if the same variants were selected as the result of parallel domestication syndromes but not if divergence was due to dissimilar domestication pressures or genetic drift (Lotterhos and Whitlock, 2015).
(4) Finally, we explored if regions of high within-species F_ST_ co-localized with regions of low F_ST_ in within-population comparisons. Δ_Div_ was used to analyze the difference between these two F_ST_ values in each window. Peaks in the Δ_Div_ statistic point to genomic regions that diverged as a result of parallel divergence from shared variation rather than due to novel variation evolving at each site (Roesti et al., 2014). As a byproduct of the Δ_Div_ calculation, the F_ST_ had to be computed in a total of eleven within-population comparisons. At the intermediate level of comparisons, F_ST_ was computed in three between-species within-genepool contrasts. These comparisons were (1) between the Andean genepool of *P. vulgaris* and the Andean I genepool of *P. lunatus*, (2) between the Mesoamerican genepool of *P. vulgaris* and the Mesoamerican I genepool of *P. lunatus*, and (3) between the Mesoamerican genepool of *P. vulgaris* and the Mesoamerican II genepool of *P. lunatus*. At the most nested level of comparisons, F_ST_ values were computed in a total of eight different within-population contrasts. For common bean there were two between-genepool wild-wild cultivated-cultivated comparisons, as follows: (1) between wild Andean and wild Mesoamerican accessions and (2) between cultivated Andean and cultivated Mesoamerican accessions. For Lima bean, F_ST_ values were calculated in three between-genepool wild-wild comparisons, as follows: (1) between wild Andean I and wild Mesoamerican I, (2) between wild Andean I and wild Mesoamerican II, and (3) between wild Mesoamerican I and wild Mesoamerican II. Also for Lima bean, F_ST_ values were estimated in three between-genepool cultivated-cultivated comparisons, as follows: (1) between cultivated Andean I and cultivated Mesoamerican I, (2) between cultivated Andean I and cultivated Mesoamerican II, and (3) between cultivated Mesoamerican I and cultivated Mesoamerican II. We refrained from calculating further single summary statistics across or within hierarchy replicates because of potential particularities in the processes behind each comparison that required individual inspection.

### Genome-Wide Patterns of Nucleotide Diversity

In order to describe patterns of diversity across the genome, we implemented a sliding window approach: window size = 1 × 10^6^bps, step size = 200 kb, already optimized for the exploration of genome-wide diversity by Cortés and Blair (2018a). We computed per-window averages of SNP density, nucleotide diversity as measured by π (Nei, 1987) and Tajima’s *D* (Tajima, 1989) using the software Tassel v.5 (Bradbury et al., 2007) and customized R scripts. Results of all windowed analyses were plotted against window midpoints in millions of base pairs (Mb) also using the software R v.3.3.1 (R Core Team). Since the distribution of the genomic islands of speciation and the within-species divergence peaks could have been constrained by genomic features, the 1 Mb flanking region of each F_ST_-based outlier window midpoint was highlighted on the same plot depicting diversity statistics across the genome. Different colors were used to distinguish among comparisons between species (*P. lunatus* vs. *P. vulgaris*), between genepools (average of four within-species between-genepools comparisons), and between domestication statuses for *P. vulgaris* (average of two within-genepool wild-cultivated comparisons) and *P. lunatus* (average of three within-genepool wild-cultivated comparisons). Means and standard errors of all three summary statistics were computed and plotted in R v.3.3.1 (R Core Team) across all types of comparisons.

## Results

### GBS Results

The raw Illumina DNA sequence data (745,927,060 high-quality barcoded reads) were processed through the GBS analysis pipeline as implemented in NGSEP (Duitama et al., 2014). The GBS analysis generated 2,000,294 unique sequence clusters (tags, Elshire et al., 2011; Glaubitz et al., 2014b). Of the total number of tags, 58.9% aligned uniquely to the *P. vulgaris* reference genome (Schmutz et al., 2014), 12.8% had multiple matches and 28.2% were unaligned. A total of 592,171 putative biallelic SNP markers were identified in the aligned tags after filtering for minimum read depth of 5X gene coverage and minimum quality score of 40. Of these, 87% with more than 20% missing data, a default threshold used for GBS studies (Glaubitz et al., 2014b), and further 59% with minimum allele frequencies (MAF) below 0.05 were excluded from the dataset. The high number of unaligned sequences and filtered markers is usual in GBS analyses (Glaubitz et al., 2014b) and in this particular case can be attributed to the naturally high levels of sequence divergence between species and genepools (Cortés and Blair, 2018a). Despite this, this GBS study yielded 22,531 SNP markers of high quality.

### Within-Species Population Structure Matched Genepool Identity and Domestication Status

**FIGURE 1:**
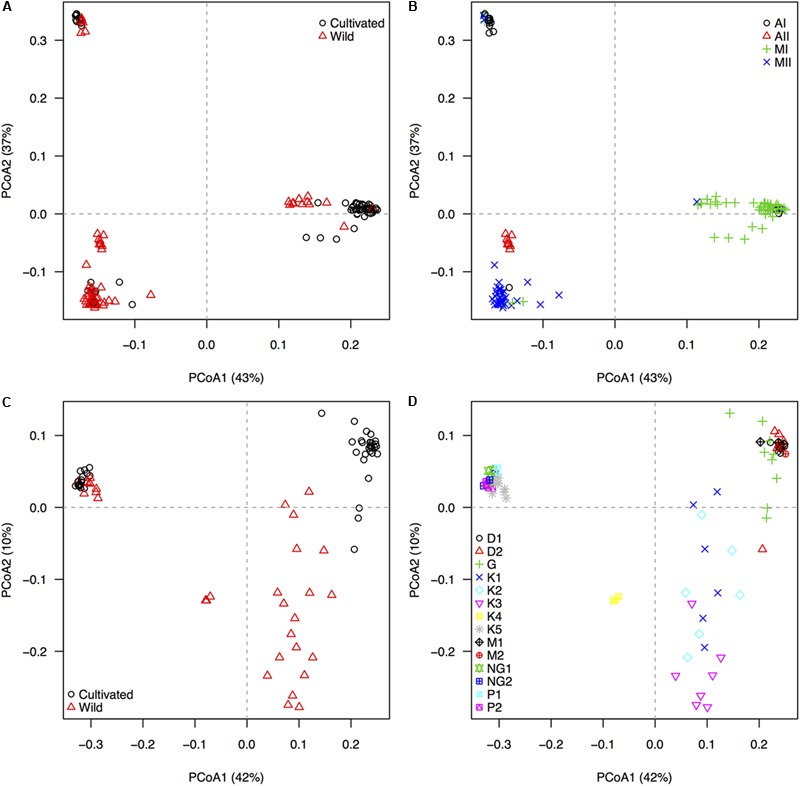
Population structure, as revealed by an unsupervised machine learning strategy implemented through a principal coordinates analyses (PCoAs), based on 22,531 GBS-derived SNP markers genotyped in 209 individuals of wild and cultivated Lima bean (*Phaseolus lunatus*) and common bean (*P. vulgaris*). First and second row of diagrams respectively display accessions of **(A,B)** *P. lunatus* and **(C,D)** *P. vulgaris*, colored by **(A,C)** domestication status and **(B,D)** genepool/race identity. Black circles and red triangles in the left panels **(A,C)** mark cultivated and wild accessions, respectively. For Lima bean **(B)**, four genepool populations, according to Chacon-Sanchez and Martinez-Castillo (2017), are indicated by different symbols. Andean genepool genotypes of *P. lunatus* are divided into subgroups AI and AII, whereas Mesoamerican genepool genotypes of *P. lunatus* are divided into subgroups MI and MII. For common bean **(D)**, nine within-genepool cultivated subpopulations, according to Blair et al. (2009), and five within-genepool wild subpopulations, according to Blair et al. (2012), are indicated by different symbols. Andean genepool genotypes of *P. vulgaris* (cloud on the upper left quadrant) are divided into cultivated subgroups NG1 and NG2 (race Nueva Granada), cultivated subgroups P1 and P2 (race Peru) and wild subgroup K5 (Andean). Mesoamerican genepool genotypes of *P. vulgaris* are divided into cultivated subgroups D1 and D2 (Durango–Jalisco complex), cultivated subgroup G (race Guatemala), cultivated subgroups M1 and M2 (race Mesoamerica), and wild subgroups K1 (Mesoamerican), K2 (Guatemalan), K3 (Colombian), and K4 (Ecuador and northern-Peru). The percentage of explained variation by each axis is shown within parenthesis in the label of the correspond axis.

The 22,531 GBS-derived SNP markers recovered, through a principal components analysis with 209 accessions, the distinctive genepool structure of *P. lunatus* and *P. vulgaris* (Figure 1). The wild-cultivated split, although noticeable, was less marked in the three main genepools of *P. lunatus* (Figure 1A) and in the Andean genepool of *P. vulgaris* (Figure 1C). Within *P. lunatus*, the Mesoamerican I genepool was as separated from the Mesoamerican II genepool as any of the Mesoamerican genepools was from the Andean I genepool (Figure 1B). The Andean II genepool was intermediate to the other three genepools, appearing slightly closer to the Mesoamerican II genepool. Within *P. vulgaris*, the Mesoamerican genepool was more scattered than the Andean genepool, mainly due to the wild Mesoamerican subpopulations that were clearly split from the cultivars. The Ecuador northern-Peru wild subpopulation was intermediate, appearing closer to the Mesoamerican wild subpopulations (Figure 1D). Race Guatemala was closer to the Mesoamerican wild subpopulations than any other Mesoamerican race.

**FIGURE 2:**
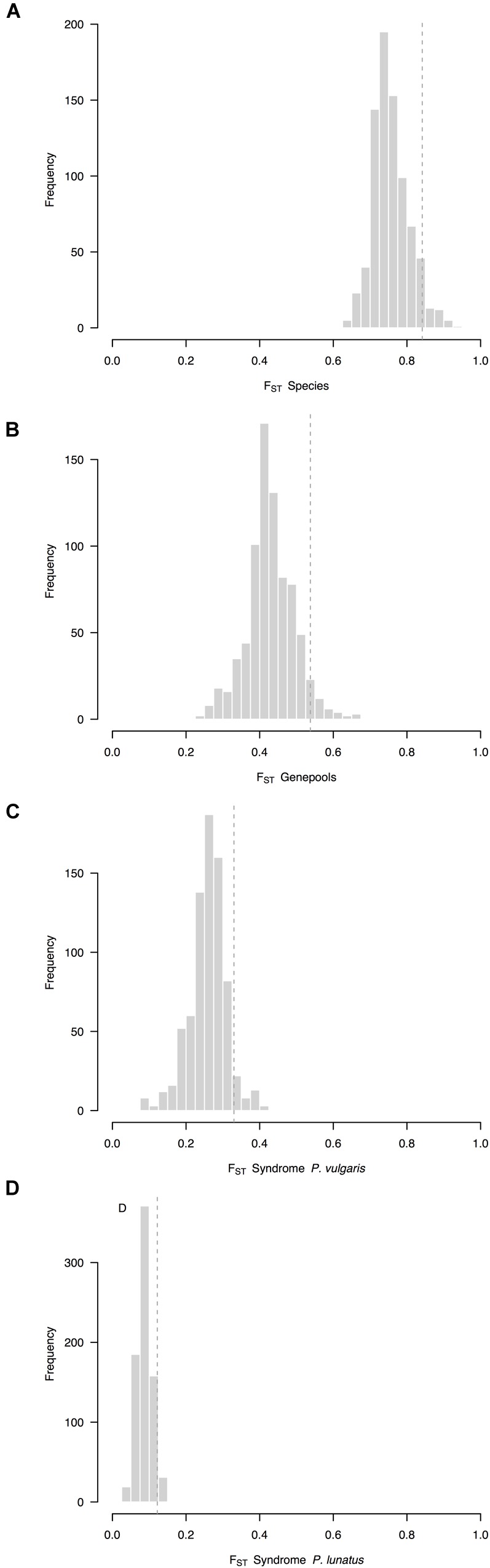
Frequency distributions of relative differentiation across hierarchically nested levels of divergence in *Phaseolus*. Relative differentiation is calculated as the fixation index (F_ST_). Average F_ST_ values per sliding window (window size = 1 × 10^7^ bps, step size = 500 kb) were computed **(A)** between species (*P. lunatus* vs. *P. vulgaris*), **(B)** between genepools (average of four within-species between-genepools comparisons), and between domestication statuses for **(C)** *P. vulgaris* (average of two within-genepool wild-cultivated comparisons, Supplementary Figures S1F,G) and **(D)** *P. lunatus* (average of three within-genepool wild-cultivated comparisons, Supplementary Figures S1H–J). Single within-species between-genepools comparisons were: Andean vs. Mesoamerican genepools of *P. vulgaris* (Supplementary Figure S1B), and Andean I vs. Mesoamerican I (Supplementary Figure S1C), Andean I vs. Mesoamerican II (Supplementary Figure S1D) and Mesoamerican I vs. II (Supplementary Figure S1E) genepools of *P. lunatus*. Dashed lines are thresholds for the identification of outliers (α = 0.05).

Overall, the average F_ST_ between species (*F*_ST_ = 0.76, CI 95%: 0.72–0.87, Figure 2A) was higher than the average F_ST_ between genepools (*F*_ST_ = 0.43, CI 95%: 0.39–0.56, Figure 2B). Both were higher than the average F_ST_ between wild and cultivated accessions of common bean (*F*_ST_ = 0.26, CI 95%: 0.24–0.36, Figure 2C), which in turn was higher than the average F_ST_ between wild and cultivated accessions of Lima bean (*F*_ST_ = 0.09, CI 95%: 0.07–0.14, Figure 2D). Within-species between-genepools comparisons were asymmetric. Although the average F_ST_ values between the Andean and Mesoamerican genepools of *P. lunatus* were indistinguishable (for the Andean I vs. Mesoamerica I comparison, *F*_ST_ = 0.61, CI 95%: 0.53–0.88, Supplementary Figure S1C; for the Andean I vs. Mesoamerica II comparison, *F*_ST_ = 0.54, CI 95%: 0.48–0.83, Supplementary Figure S1D), both of them were higher than the average F_ST_ values between the Mesoamerican genepools of *P. lunatus* (*F*_ST_ = 0.35, CI 95%: 0.31–0.52, Supplementary Figure S1E) and between the Andean and Mesoamerican genepools of *P. vulgaris* (*F*_ST_ = 0.22, CI 95%: 0.18–0.42, Supplementary Figure S1B). Within-genepool wild-cultivated comparisons were also asymmetric. The average F_ST_ between wild and cultivated accessions of Andean common bean (*F*_ST_ = 0.37, CI 95%: 0.35–0.44, Supplementary Figure S1F) exceed the average F_ST_ between wild and cultivated accessions of Mesoamerican common bean (*F*_ST_ = 0.15, CI 95%: 0.10–0.30, Supplementary Figure S1G). Both comparisons surpassed the average F_ST_ values between wild and cultivated accessions within genepools of Lima bean, which were equivalent among them (within the Andean I genepool, *F*_ST_ = 0.05, CI 95%: 0.03–0.13, Supplementary Figure S1H; within the Mesoamerican I genepool, *F*_ST_ = 0.12, CI 95%: 0.10–0.20, Supplementary Figure S1I; within the Mesoamerican II genepool, *F*_ST_ = 0.09, CI 95%: 0.08–0.17, Supplementary Figure S1J).

### The Between-Species and Within-Species Genomic Divergence Profiles Overlapped Scatteredly

**FIGURE 3:**
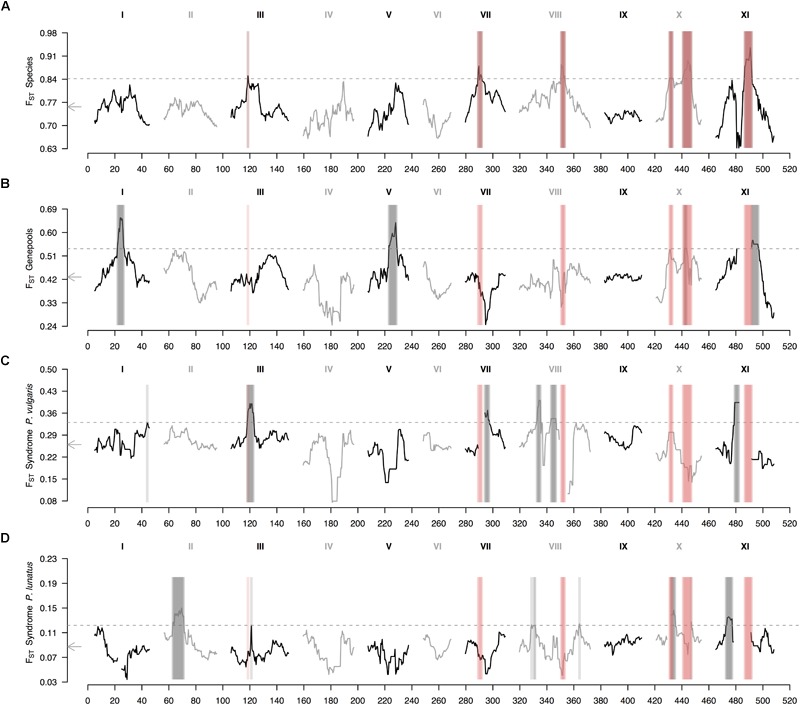
Genomic landscape of relative differentiation in *Phaseolus* across hierarchically nested levels. Sliding window analyses (window size = 1 × 10^7^ bps, step size = 500 kb) for relative differentiation, as measured by the fixation index (F_ST_), were done **(A)** between species (*P. lunatus* vs. *P. vulgaris*), **(B)** between genepools (average of four within-species between-genepools comparisons, Supplementary Figures S2B–E), and between domestication statuses for **(C)** *P. vulgaris* (average of two within-genepool wild-cultivated comparisons, Supplementary Figures S2F,G) and **(D)** *P. lunatus* (average of three within-genepool wild-cultivated comparisons, Supplementary Figures S2H–J). Original within-species between-genepools comparisons are: Andean vs. Mesoamerican genepools of *P. vulgaris* (Supplementary Figure S2B), and Andean I vs. Mesoamerican I (Supplementary Figure S2C), Andean I vs. Mesoamerican II (Supplementary Figure S2D) and Mesoamerican I vs. II (Supplementary Figure S2E) genepools of *P. lunatus*. Results of all windowed analyses are plotted against window midpoints in millions of base pairs (Mb). Black and gray colors highlight different common bean chromosomes according to Schmutz et al. (2014). Gray arrows on the vertical axes mark genome-wide averages. Gray dashed horizontal lines indicate F_ST_ thresholds for the identification of outliers (from Figure 2). Vertical translucent boxes feature the 1 Mb flanking region of each outlier window midpoint for between- (red boxes) and within- (gray boxes) species comparisons.

The genomic landscape of species divergence revealed six outlier regions in chromosomes Pv3, Pv7, Pv8, Pv10, and Pv11 (Figure 3A). Chromosome Pv10 exhibited two outlier regions split by a ‘high valley.’ On the other hand, the average genomic landscape of genepool divergence revealed four outlier regions in chromosomes Pv1, Pv5, Pv10, and Pv11 (Figure 3B). The region in chromosome Pv10 overlapped with a between-species peak, whereas the region in chromosome Pv11 fell beside a between-species outlier region. Finally, the average genomic landscape of domestication revealed six outlier regions in common bean (Figure 3C) and seven outlier regions in Lima bean (Figure 3D), of which only one was shared (in chromosome Pv3). Of these eleven different regions, only three, in chromosomes Pv3 and Pv10, overlapped to outlier regions in the between-species comparisons.

When exploring in more detail the genomic landscape of relative differentiation in *Phaseolus* beans at different hierarchically nested levels, parallel divergence among between-species and within-species comparisons remained limited (Supplementary Figure S2). The only between-species divergence peak that reappeared consistently in individual between-genepools comparisons was the one in chromosome Pv11 (Supplementary Figures S2B–D). This same region was outlier in the wild vs. cultivated F_ST_ profile of Mesoamerican I Lima bean (Supplementary Figure S2I). Similarly, the only between-species divergence peak that reappeared somehow steadily in individual wild-cultivated comparisons was the one in chromosome Pv3 (Supplementary Figures S2F,G), which was recovered in the F_ST_ profiles of both common bean domestications (Supplementary Figures S2F,G), and was adjacent to an outlier region in the F_ST_ profile of the Andean I Lima bean domestication (Supplementary Figure S2H). Also recurrent across profiles, with less consistency though, was the between-species divergence region in chromosome Pv10 that coincided with F_ST_ peaks in all between-genepools comparisons of *P. lunatus* (Supplementary Figures S2C–E), except for the Andean I vs. Mesoamerican II contrast (Supplementary Figure S2D), as well as in the domestication profiles of Andean common bean (Supplementary Figure S2F), and Andean I (Supplementary Figure S2H) and Mesoamerica II Lima bean (Supplementary Figure S2J).

### Within-Species Divergence Revealed Some Signatures of Parallelism and Shared Variation

Within-species between-genepools divergence F_ST_ profiles (Supplementary Figures S2B–E) were partially similar among the four available comparisons (Andean vs. Mesoamerican genepools of *P. vulgaris*, and Andean I vs. Mesoamerican I, Andean I vs. Mesoamerican II and Mesoamerican I vs. Mesoamerican II genepools of *P. lunatus*). Peaks in chromosomes Pv1 and Pv11 reappeared in all four comparisons, although the peak in chromosome Pv11 was slightly shifted leftward in the comparison between the Mesoamerican genepools of *P. lunatus* (Supplementary Figure S2E), so that it did not overlap the between-species peak that the other comparisons did. This same comparison lacked a peak in chromosome Pv5 that was consistently observed in the other three comparisons (Supplementary Figures S2B–D), and exhibited an outlier peak in chromosome Pv10 that was also detected in the Andean I vs. Mesoamerican I comparison within Lima bean (Supplementary Figure S2C), overlying a between-species peak. Single peaks in chromosomes Pv2 and Pv8, and in chromosomes Pv4 and Pv6, respectively appeared only once in that comparison (Mesoamerican genepools of *P. lunatus*, Supplementary Figure S2E) and in the between-genepools comparison within common bean (Supplementary Figure S2B).

Within-genepool wild-cultivated divergence F_ST_ profiles (Supplementary Figures S2F–J) were also moderately recurrent across all five available comparisons (wild vs. cultivated *P. vulgaris* within the Andean and Mesoamerican genepools, and wild vs. cultivated *P. lunatus* within the Andean I, Mesoamerican I and Mesoamerican II genepools). A couple of peaks re-appeared in several profiles of the domestication syndrome and overlapped differentiated regions between species. The first one, a divergence peak in chromosome Pv3, was repeatedly retrieved in the genomic profiles of the common bean domestications (Supplementary Figures S2F,G) and the Lima bean Andean I domestication (Supplementary Figure S2H). Likewise, an outlier region at the beginning of chromosome Pv10 was recurrently recovered in the genomic profiles of the Andean domestications of both species (Supplementary Figures S2F,H) and the Lima bean Mesoamerican II domestication (Supplementary Figure S2J). Among the few consistent signatures of domestication, a divergence peak in chromosome Pv11 re-appeared in the genomic profiles of all Mesoamerican syndromes (Supplementary Figures S2G,I,J). Toward the left of this region, another shared outlier F_ST_ peak in chromosome Pv11 was detected in the Andean I (Supplementary Figure S2H) and the Mesoamerican I (Supplementary Figure S2I) domestication profiles of Lima bean. These two peaks were separated by a ‘high valley’ and fell beside the outlier peak detected for all within-species between-genepools comparisons (Supplementary Figures S2B–E), which coincided with a between-species peak (Supplementary Figure S2A) only recovered by the profile of the Mesoamerican I Lima bean domestication (Supplementary Figure S2I). An outlier region also captured in all F_ST_ profiles of the Mesoamerican domestications (Supplementary Figures S2G,I), except for the one of the Mesoamerican II Lima bean (Supplementary Figure S2J), was located in chromosome Pv8. At the beginning of the same chromosome, three outlier peaks almost overlapped between both common bean domestications (Supplementary Figures S2F,G) and the Lima bean Mesoamerican II domestication (Supplementary Figure S2J). All outlier windows in chromosome Pv8 flanked a between-species divergence peak (Supplementary Figure S2A). Also tangentially, a peak in chromosome Pv2 observed in the Mesoamerican common bean domestication F_ST_ profile (Supplementary Figure S2G) re-appeared in the profiles of all the Lima bean domestications (Supplementary Figures S2H–J). Single outlier peaks in chromosomes Pv4 and Pv7 were respectively detected for the Andean (Supplementary Figure S2F) and the Mesoamerican (Supplementary Figure S2G) domestications of common bean, and in chromosomes Pv1 and Pv4 for the Andean domestication of Lima bean (Supplementary Figure S2H).

**FIGURE 4:**
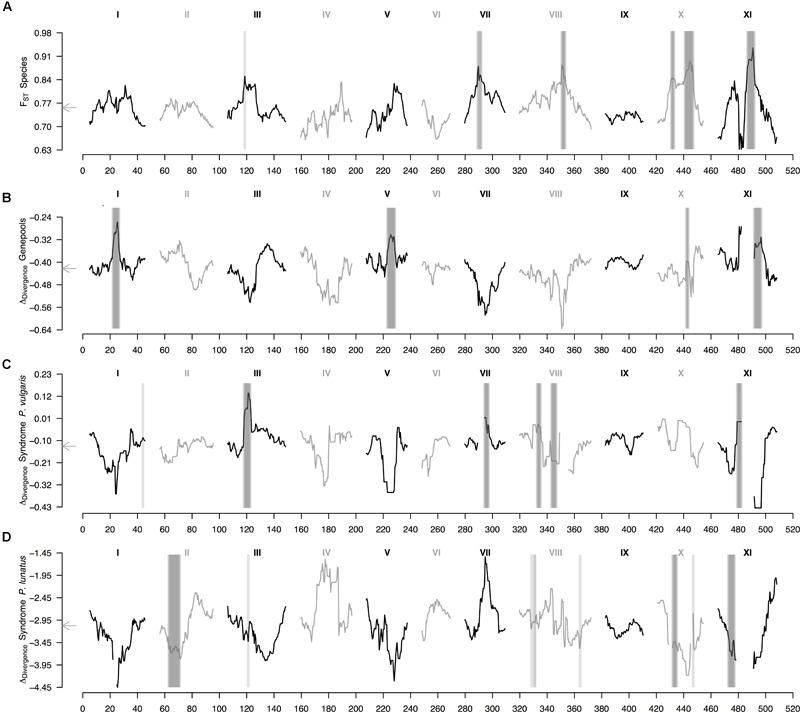
Genomic landscape of divergence in *Phaseolus* beans across hierarchically nested levels. Sliding window analyses (window size = 1 × 10^7^ bps, step size = 500 kb) are shown for: **(A)** relative differentiation (from Figure 3A) computed as the fixation index (F_ST_) between species (*P. lunatus* vs. *P. vulgaris*), **(B)** delta divergence (Δ_Div_), according to Roesti et al. (2014), between genepools (results from the average F_ST_ of four within-species between-genepools, Supplementary Figures S2B–E, and three between-species within-genepool, Supplementary Figures S4A–C, comparisons), **(C)** Δ_Div_ between domestication statuses for *P. vulgaris* (results from the average F_ST_ of two within-genepool wild-cultivated comparisons, Supplementary Figures S2F,G, and two between-genepool wild-wild cultivated-cultivated comparisons, Supplementary Figures S4D,E) and **(D)** Δ_Div_ between domestication statuses for *P. lunatus* (results from the average F_ST_ of three within-genepool wild-cultivated comparisons, Supplementary Figures S2H–J, and six between-genepool wild-wild cultivated-cultivated comparisons, Supplementary Figures S4F–K). Results of all windowed analyses are plotted against window midpoints in millions of base pairs (Mb). Black and gray colors highlight different common bean chromosomes according to Schmutz et al. (2014). Gray arrows on the vertical axes mark genome-wide averages. Vertical translucent gray boxes highlight the 1 Mb flanking region of each F_ST_-based outlier window midpoint (from Figure 3).

Finally, the within-species Δ_Div_ statistic (Figure 4 and Supplementary Figure S3), indicative of parallel divergence from shared variation, revealed that regions of high F_ST_ generally co-localized with regions of low F_ST_ in within-population comparisons (Supplementary Figure S4). This pattern mostly held for the average landscape of genepool divergence, for which all within-species between-genepools differentiated regions overlapped peaks in the Δ_Div_ profile (Figure 4B); as well as for the average landscape of domestication in common bean, for which five out of six wild-cultivated differentiated regions overlaid peaks in the Δ_Div_ profile (Figure 4C, the exception was the region in the tail of chromosome Pv8). However, the average landscape of domestication in Lima bean did not have any wild-cultivated differentiated region intersecting a peak in the Δ_Div_ profile (Figure 4D), meaning that in this case domestication likely did not involve as much shared variation than for common bean. The outlier region in chromosome Pv3 that was shared by the two average wild-cultivated F_ST_ profiles intersected a Δ_Div_ peak for the domestication of common bean (Figure 4C) but not for the domestication of Lima bean (Figure 4D).

### Genomic Islands Correlated With Intrinsic Genomic Features

**FIGURE 5:**
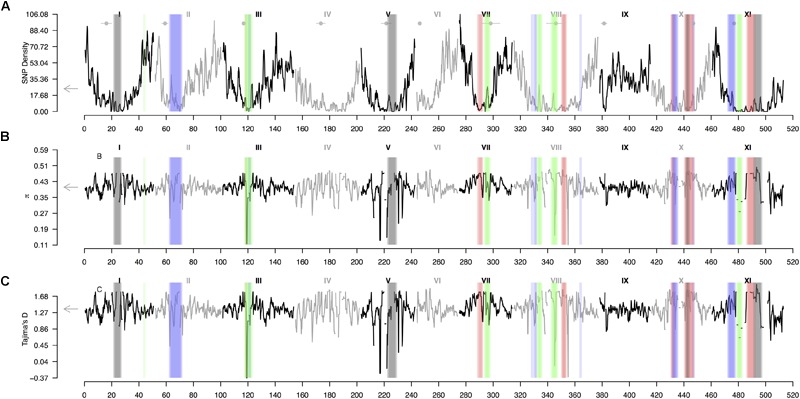
Patterns of genome-wide diversity in common bean and Lima beans. A sliding window analysis (window size = 1 × 10^6^ bp, step size = 200 kb) was used to compute **(A)** SNP density, **(B)** nucleotide diversity as measured by π, and **(C)** Tajima’s *D*. Vertical translucent boxes highlight the 1 Mb flanking region of each F_ST_-based outlier window midpoint (from Figure 3) when F_ST_ was computed as follows: (red boxes, from Figure 3A) between species (*P. lunatus* vs. *P. vulgaris*), (gray boxes, from Figure 3B) between genepools (average of four within-species between-genepools comparisons, Supplementary Figures S2B–E), (green boxes, from Figure 3C) between domestication statuses for *P. vulgaris* (average of two within-genepool wild-cultivated comparisons, Supplementary Figures S2F,G) and (blue boxes, from Figure 3D) between domestication statuses for *P. lunatus* (average of three within-genepool wild-cultivated comparisons, Supplementary Figures S2H–J). Results of all windowed analyses are plotted against window midpoints in millions of base pairs (Mb). Black and gray colors highlight different common bean (Pv) chromosomes. Gray arrows on the vertical axes indicate genome-wide averages. Horizontal gray lines with a central filled gray dot at the top of the figure mark the centromeres (from Schmutz et al., 2014).

A sliding window analysis (window size = 1 × 10^6^ bp, step size = 200 kb) was used to explore the patterns of genome-wide diversity. Marker density decayed drastically toward the centromeres. Average marker density was 24 SNPs per million base pairs (95% CI, 7–73, Figure 5A). Average nucleotide diversity as measured by π was 0.40 per million base pairs (95% CI, 0.38–0.47, Supplementary Figure 5B). Average Tajima’s *D* was 1.36 per million base pairs (95% CI, 1.24–1.79, Figure 5C). High values of π and Tajima’s *D* mirrored strong hierarchically nested population structure.

**FIGURE 6:**
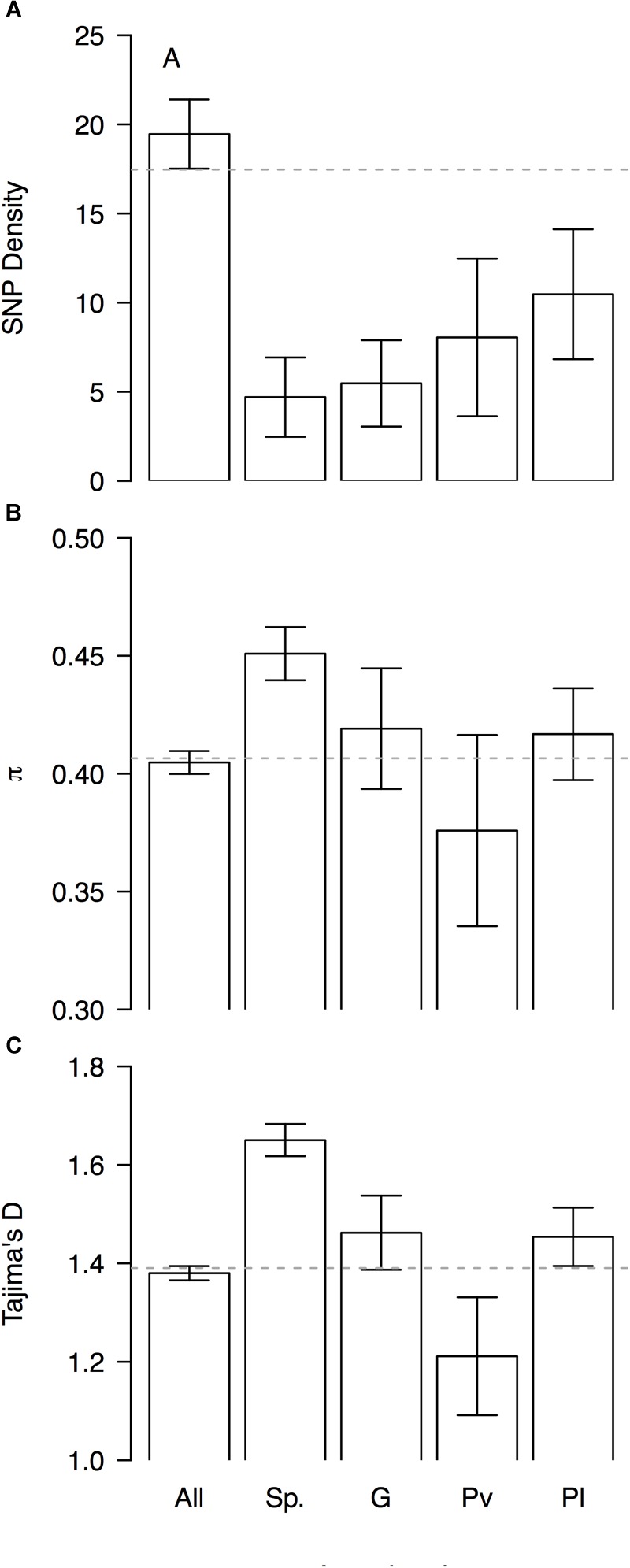
Patterns of genome-wide diversity in genomic windows significantly differentiated across hierarchically nested levels of divergence. Means and standard errors (from Figure 5) are shown for **(A)** SNP density, **(B)** nucleotide diversity as measured by π, and **(C)** Tajima’s *D*, when computed in (‘All’) the entire set of possible sliding windows (window size = 1 × 10^6^ bp, step size = 200 kb), and only windows that significantly diverged (‘Sp.’) between species (*P. lunatus* vs. *P. vulgaris*, Figure 2A), (‘G’) between genepools (average of four within-species between-genepools comparisons, Figure 2B), and between domestication statuses for (‘Pv’) *P. vulgaris* (average of two within-genepool wild-cultivated comparisons, Figure 2C) and for (‘Pl’) *P. lunatus* (average of three within-genepool wild-cultivated comparisons, Figure 2D). Gray dashed horizontal lines mark genome-wide averages.

Genomic differentiation across hierarchically nested scales of divergence always coincided with regions of relatively low SNP density regardless the nature of the comparison (average SNP density for all windows 19 ± 2 vs. divergent windows between: species 5 ± 4, genepools 5 ± 4, and domestication syndromes in *P. vulgaris* 8 ± 4 and in *P. lunatus* 10 ± 4, Figure 6A). However, the F_ST_-based outlier regions spanned a wide range of π values (all windows 0.405 ± 0.005 vs. divergent windows between: species 0.45 ± 0.01, genepools 0.42 ± 0.03, and domestication syndromes in *P. vulgaris* 0.38 ± 0.04 and in *P. lunatus* 0.42 ± 0.02, Figure 6B) and Tajima’s *D* values (all windows 1.38 ± 0.01 vs. divergent windows between: species 1.65 ± 0.03, genepools 1.46 ± 0.08, and domestication syndromes in *P. vulgaris* 1.2 ± 0.1 and in *P. lunatus* 1.45 ± 0.06, Figure 6C). The latter were significantly inflated in divergent windows between species and reduced in divergent windows between wild and cultivated common bean.

## Discussion

We have found that genomic islands of speciation are not necessarily more prone to harbor within-species divergence, yet subjacent genomic constrains could still be shaping parallel divergence at broader genomic scales. With that in mind, we first discuss some evidence of parallelisms in the genetic adaptations to the Mesoamerican and Andean environments in common and Lima beans resulting from shared variation. Later, we review cases of moderate repeatability in the genomic consequences of multiple domestication events. Finally, we argue that despite those genetic variants may have been recruited in parallel by selective processes alone, genomic features and linked selection could have also enhanced convergent differentiation in low-recombining regions. Our study shows that differentiation across repeated and hierarchically nested levels of divergence always co-occurs with regions of low SNP density, and in some cases with chromosomal rearrangements. These concurring signatures may be a byproduct of genomic constrains inherent to low-recombining regions and intrinsic genomic features. We advise a more systematic use of repeated and hierarchically nested samplings in order to improve our understanding of the underlying causes of the genomic landscape of divergence.

### Genomic Islands of Speciation Are Not More Prone to Harbor Within-Species Divergence

We predicted that if genomic islands of speciation in *Phaseolus* species were more prone to harbor within-species divergence due to reduced recombination and increased drift, then between-species F_ST_ outliers should be recovered in within-species comparisons. Of the six different speciation islands found in this study, only three, in chromosomes Pv3 and Pv10, are shared with any of the 11 regions found as outliers in the within-species comparisons, leading to an overlap of 21%. Parallel divergence among between-species and within-species comparisons remains limited when exploring in more detail the genomic landscape of relative differentiation in *Phaseolus* beans at different hierarchically nested levels. Recurrent across profiles is the between-species divergence region in chromosome Pv10, where a pericentric inversion between species is reported (Bonifácio et al., 2012) – a genomic feature that we do not discard could enhance divergence at other regions as well (i.e., Pv11). In addition, the only between-species divergence peak that reappears consistently in individual between-genepools comparisons is the one in chromosome Pv11, whereas the only between-species divergence peak that reappears somehow steadily in individual wild-cultivated comparisons is the one in chromosome Pv3. Therefore, we can conclude that between-species F_ST_ outliers in *Phaseolus* beans are not always recovered in within-species comparisons and that within-species divergence does not necessarily arises within the speciation islands. At first glance, this result could discard the phantom of spurious processes pushing up the genomic islands of speciation (Pennisi, 2014; Ellegren and Wolf, 2017; Ravinet et al., 2017). However, as discussed in the last section of the discussion, subjacent genomic constrains may still shape parallel divergence at a broader scale across the genome.

### Within-Species Parallel Divergence Partially Results From Shared Variation

Our study provides evidence of some putative parallelisms in the genetic adaptations to the Mesoamerican and Andean environments in common and Lima beans. This finding assumes that there has been neglectful gene flow between cultivars and does not discard that genomic features can still play a role in shaping within-species genomic divergence. Of the eight outlier regions in the within-species between-genepools divergence F_ST_ profiles, three peaks (37%) in chromosomes Pv1, Pv5, and Pv11 reappear somehow consistently across all four comparisons. Besides this exploration, the landscape of genomic adaptation has remained largely unexplored in *Phaseolus* beans. Among the few other studies addressing this question, a panel of wild common bean sampled across the Andean and Mesoamerican ranges revealed that regardless the strength of the bottlenecks (Ariani et al., 2017), the signatures of divergent adaptation are widespread along the genome and coincided with regions of elevated SNP density (Cortés and Blair, 2018a), frequent recombination and high gene content (Blair et al., 2018). However, theses surveys have not explicitly addressed the colonization of the Andes by lineages coming from Central America and the corresponding change in selection pressures associated with different altitudes, latitudes and micro-environments. Topographically complex mountainous systems, such as the Andes, harbor an impressive heterogeneity of climates at a small scale (Cortés and Blair, 2018b; Cortés and Wheeler, 2018). The ridges and valleys constitute physical barriers that can limit dispersal and cause local variation in rainfall, resulting in genetic isolation and variation in habitats (Cortés et al., 2014; Sedlacek et al., 2014, 2015, 2016; Wheeler et al., 2014, 2015, 2016; Little et al., 2016). Both processes have likely speeded up the evolution of high species diversity in this region (Antonelli et al., 2009; Hoorn et al., 2010; Madriñán et al., 2013; Arnegard et al., 2014; Cortés et al., 2018). Yet, the relative effects of geographic isolation, environmental variation at a small scale, and their potential interactions across genepools remain poorly understood in wild beans. Characterizing in more detail the genomic consequences associated with the colonization of diverse habitats may disclose new cases of genetic parallelism in the adaptation of beans.

The genomic consequences of multiple domestication events are also moderately recurrent as revealed by our survey. From the twelve regions putatively differentiated as the result of the domestication syndrome, only 5 (42%) appear in more than one comparison but none appears in all. Two peaks in chromosome Pv3 and Pv10 are repeated across three different comparisons of all five profiles of the domestication syndromes. At least the region in chromosome Pv3 has been reported to be involved in the vernalization pathway (i.e., *Phvul.003G033400*) as part of the Mesoamerican domestication of common bean, as well as with seed growth (Schmutz et al., 2014). Two other divergence peaks in chromosome Pv8 and Pv11 are consistent across all three genomic profiles of the Mesoamerican domestication syndrome. The region in chromosome Pv8 is known for being related with the encoding of the nitrate reductase (i.e., *Phvul.008G168000*), a critical element for plant and seed growth, during the Mesoamerican domestication of common bean (Schmutz et al., 2014). Also as part of this domestication event, the region in chromosome Pv11 is associated with increased plant size through the ubiquitin ligase degradation pathway (i.e., *Phvul.011G213300*) that controls flower and stem size (Schmutz et al., 2014). More loosely, a peak at chromosome Pv2 in the Mesoamerican common bean domestication F_ST_ profile is recovered in the profiles of all three Lima bean domestications. This region has been linked with the domestication syndrome of Lima bean since it is involved in the regulation of seed germination (i.e., *Phvul.002G033500*) and leaf size (i.e., *Phvul.002G041800*) and is enriched by inflated linkage disequilibrium scores (Chacon-Sanchez and Martinez-Castillo, 2017). Also, a QTL (St) related with dehiscence has been reported in this region, but candidate genes are still unknown (Koinange et al., 1996). Although scattered, some of these few regions may reveal truth parallelisms in the domestication syndromes, whereas others may still be constrained by genomic features.

Also striking is the rarity of regions putatively involved in domestication and shared by several domestication events. This trend, mostly expected for quantitative traits with complex genetic architectures (Blair et al., 2006; Meyer et al., 2012) because of the complexity in the interaction of selection, drift, novel mutations and epistatic effects (Chevin and Hospital, 2008; Csillery et al., 2018), had already been noticed for common bean (Schmutz et al., 2014) – potentially applying for Lima bean as well (Chacon-Sanchez and Martinez-Castillo, 2017), and so does not necessarily speak for a prevalent role of drift. Yet, since divergence in the lack of repeatability is still a liable result of lineage sorting, caution must be undertaken while interpreting these signals. Single outlier regions for the common bean Andean–Mesoamerican split and the Lima bean Mesoamerican I–Mesoamerican II split are found in chromosomes Pv4 and Pv6, and in chromosomes Pv2 and Pv8, respectively. Similarly, single outlier peaks associated with the domestication of the Andean and Mesoamerican common bean and the Andean Lima bean are respectively found in chromosomes Pv4, Pv7, and Pv1 and Pv4. These eight singularities may result from different adaptive pressures across the Americas unique to each species, distinctive adaptation to the Mesoamerican micro-environments, dissimilar selection as part of each domestication event (Ravinet et al., 2017), equivalent selective forces acting on different genetic variants (Roesti et al., 2014; Ravinet et al., 2016), or genetic drift (Lotterhos and Whitlock, 2015). Discerning among these causes may require further genotyping in an extended panel specifically addressing each comparison. At least for the divergence peak at chromosome Pv7 in the wild-cultivated Mesoamerican common bean comparison, other drivers besides the domestication itself are an unlikely reason for divergence because a wide region in chromosome Pv7 region is known for being associated with increased seed weight (i.e., *Phvul.007G094299* – *Phvul.007G.99700*) during the Mesoamerican domestication of common bean (Schmutz et al., 2014), as well as with flowering regulation (i.e., *Phvul.007G096500* and *Phvul.007065600*) as part of the domestication of Lima bean (Chacon-Sanchez and Martinez-Castillo, 2017) and both common bean genepools (Schmutz et al., 2014).

The origin of the variants exhibiting parallel divergence from novel or standing genetic variation leads to distinctively different patterns of genomic divergence (Hermisson and Pennings, 2005; Barrett and Schluter, 2008; Pritchard et al., 2010). The Δ_Div_ statistic is indicative of parallel divergence from shared variation and reveals that regions of high F_ST_ for the within-species comparisons generally co-localized with regions of low F_ST_ in within-population comparisons. This pattern mostly holds for the average landscape of genepool divergence and for the average landscape of domestication in common bean and indicates a predominance of parallel divergence from shared variation rather than due to novel variation evolving at each site (Roesti et al., 2014). The average landscape of domestication in Lima bean, however, only has one out of seven wild-cultivated differentiated regions intersecting a peak in the Δ_Div_ profile. This peak, in the same tail of chromosome Pv7 that has been associated with multiple domestication syndromes as discussed in the previous paragraph, may therefore also be the result of divergence from shared variation. Despite that shared variation may have been recruited in parallel by selective processes alone, genomic features and linked selection could have also enhanced convergent differentiation (Wolf and Ellegren, 2016; Ellegren and Wolf, 2017), as discussed below.

### Genomic Features Constrain Divergence Across Scales

Our study shows that differentiation across repeated and hierarchically nested levels of divergence always co-occurs with regions of low SNP density. Increased lineage sorting, and consequently rapid differentiation, is a common phenomenon in low-recombining regions because of linked selection and a reduction in the effective population size (Jones et al., 2012b; Zhou et al., 2014; Wolf and Ellegren, 2016). Likewise, low-recombining regions also tend to exhibit a decline in diversity due to background selection and, to a lower extent, because of genetic hitchhiking (Ellegren and Galtier, 2016; Martin and Jiggins, 2017; Ma et al., 2018; Martin et al., 2018). In our study we have found evidence that regions with low SNP diversity are enriched for contiguous signatures of differentiation between bean species, between genepools and as part of the multiple domestication syndromes. These concurring signatures may be a byproduct of genomic constrains inherent to low-recombining regions.

One of the regions that repeatedly exhibit high differentiation across hierarchically nested levels of divergence in the presence of low SNP density was the centromeric section of chromosome Pv11. The wild-cultivated divergence peak in this chromosome is shared by three domestication syndromes and is located beside the outlier peak detected for all within-species between-genepools comparisons, which in turn coincides with a major between-species peak. In this wide section of chromosome Pv11 our analysis further revealed that convergent divergence is consistently correlated with very low SNP density, as expected because of combined effects of linked and background selection in low-recombining regions (Jones et al., 2012b; Zhou et al., 2014; Wolf and Ellegren, 2016; Ravinet et al., 2017). The observation that genomic constrains are biasing divergence across scales in this section of chromosome Pv11 is reinforced by the fact that previous genomic scans did not attribute to this region a consisted outstanding role during the domestication syndromes (Schmutz et al., 2014; Chacon-Sanchez and Martinez-Castillo, 2017) or in conferring adaptation to different environments and latitudes across the Americas (Cortés et al., 2012a,b, 2013; Blair et al., 2016; Cortés and Blair, 2018a). The only exception is the candidate gene influencing plant size (*Phvul.011G213300*) as part of the Mesoamerican domestication syndrome of common bean (Schmutz et al., 2014), but then this pattern has not been consistently reported for the other domestication events as to explain its steady repeatability across hierarchically nested levels of divergence in windows with low SNP density.

Other ‘hotspots’ for spurious divergence due to genomic constrains may be the regions with low SNP density in chromosomes Pv8 and Pv10 that exhibit signatures of between-species divergence as well as repeated between genepools and within-genepool wild-cultivated divergence. The region in chromosomes Pv8 was previously reported to be highly divergent during the domestication of the Andean common bean, but then there were not candidate genes in this region associated with that domestication syndrome in particular (Schmutz et al., 2014), despite that the same region is known for being involved in plant and seed growth (i.e., *Phvul.008G168000*) during the Mesoamerican domestication of the same species. This paradox may then be a consequence of genomic constrains obscuring genuine anthropic selection and repeatedly forcing divergence in this region. Similarly, the wide divergent region in chromosomes Pv10, characterized by two outlier peaks split by a ‘high valley,’ actually matches a pericentric inversion between species (Bonifácio et al., 2012), exemplifying how genomic features inexorably condition differentiation across scales of divergence. This coincidence unlikely is an artifact in the genome assembly because both available versions of the reference genome are equally robust for mapping GBS reads and detecting the same overall F_ST_ estimates regardless reported inversions (Supplementary Figure S5).

The observation that low-recombining regions are enriched for differentiation across repeated and hierarchically nested levels of divergence in *Phaseolus* beans opposes the profiles of the genome-wide selection scans carried out in common bean. While low-recombining regions are more prone to exhibit signatures of divergence, regions toward the arms of the chromosomes with high SNP density more often harbor adaptive variation (Cortés and Blair, 2018a). This trend follows expectations because low-recombining regions are more liable to display divergence because of linked selection (Ellegren et al., 2012; Ellegren and Galtier, 2016; Ellegren and Wolf, 2017), whereas recombination hotspots usually exhibit higher SNP density and are enriched with functional genes (Rodgers-Melnick et al., 2015; Ellegren and Galtier, 2016) – an already well-described relationship for common bean (Bhakta et al., 2015; Blair et al., 2018). Also, adaptive divergent selection usually homogenizes haplotypes within the same niche and fixes polymorphisms in different populations, so that few haplotypes with high frequency remain. This selective process leads to high values of nucleotide diversity and Tajima’s *D*, and low values of the Watterson’s theta (𝜃) estimator (Wakeley, 2008), a tendency that was corroborated in wild common bean when looking for adaptive variants (Cortés and Blair, 2018a) but that was lacking in the present study while retrieving the genomic landscape of divergence between species, genepools and domestication statuses.

In short, our study provides comprehensive evidence that, despite some genuine parallelism accounting for the recruitment of shared variants at replicated comparisons in the between-genepools comparisons and as part of the domestication syndromes, the consequences of intrinsic genomic features are compelling across different hierarchically nested levels of divergence. Because certain regions are more prone to accumulate islands of divergence as the result of genomic constrains, we advocate that studies of genomic divergence should consider more systematically a dual-purpose sampling, such as ours. First, using replicated populations under presumably similar selection pressures helps accounting for lineage sorting and characterizing the nature of the selected variants – i.e., novel vs. standing (Roesti et al., 2014). Second, a hierarchically nested sampling across various levels of divergence allows for further assessments on the processes, that like genomic constrains, may give rise to parallel divergence patterns (Hermisson and Pennings, 2005; Barrett and Schluter, 2008; Pritchard et al., 2010; Pereira et al., 2016). Finally, some of these examinations must be verified with genomic features and estimates of the recombination rate (Maynard Smith and Haigh, 1974; Nielsen, 2005; Storz, 2005). Ultimately, our work could be seen as the first exploration of this combined sampling tested in two *Phaseolus* bean species that exhibit strong genepool structure and multiple domestication events. We foresee that as the evidence of pervasive genomic constrains shaping genomic differentiation across species at countless scales of divergence accumulates, replicated samplings of contrasting populations in a hierarchically nested framework of divergence will become indispensible.

## Perspectives

The putatively adaptive divergence regions identified in this study must be validated. Similar selective pressures at the Mesoamerican and the Andean regions could account for some of the parallelisms in the between-genepools divergence profiles. Similarly, parallel domestication pressures acting on the same genetic variants could be the leading cause for some of the parallelisms observed in the wild-cultivated divergence profiles that were not biased by genomic constrains. A denser genotyping, e.g., by whole genome sequencing – WGS s. Lobaton et al. (2018), in a panel of contrasting genotypes specifically addressing key comparisons would allow narrowing these divergence peaks and identifying candidate pathways (Fuentes-Pardo and Ruzzante, 2017). Pursuing this research is crucial because understanding the genomic signatures of adaptation and domestication is useful for germplasm characterization and offers the potential to enhance breeding by exploiting naturally available genetic variants. On the other hand, among the five domesticated species in the *Phaseolus* genus, common and Lima beans are the only ones exhibiting range expansions toward South American and multiple domestications (Bitocchi et al., 2017). However, exploring the landscape of divergence in other domesticated *Phaseolus* species is equally insightful because of their overlapping distribution ranges, nested phylogenetic relationships and divergent adaptations. For instance, Year (*P. dumosus*) and Runner (*P. coccineus*) beans are Mesoamerican and well adapted to humid habitats, which makes them a potential source of resistance to biotic stresses. On the other hand, Tepary bean (*P. acutifolius*) is also Mesoamerican but is well known for growing in desert and semi-arid environments, which makes it a likely source of tolerance to abiotic stresses. These species also possess well-established genomic resources (Guerra-Garcia et al., 2017) that could speed up newer genome-wide comparisons. *Phaseolus* species that never underwent domestication are also abundant (ca. 70) and could enrich our understanding of genomic divergence in this intricate genus. Considering all *Phaseolus* species will ultimately reinforce beans as a model for understanding speciation, adaptation and crop evolution (Broughton et al., 2003; Cortés, 2013; Bitocchi et al., 2017).

Extensive samplings meant to validate putatively adaptive divergence regions and to better characterize the genomic landscape of divergence in all *Phaseolus* beans species could benefit from explicit comparisons of the profiles of relative (F_ST_) and absolute (D_XY_) between-population divergence. F_ST_ vs. D_XY_ comparisons can inform about population divergence in the presence of gene flow (co-occurrence of peaks in both profiles), recurrent selection across subpopulations (co-occurrence of F_ST_ peaks with shallow D_XY_ valleys) and selective sweeps predating the subpopulations’ split (co-occurrence of F_ST_ peaks with deep D_XY_ valleys) (Nachman and Payseur, 2012; Cruickshank and Hahn, 2014; Irwin et al., 2016). Discerning among these processes will improve our understanding of the range expansions and the multiple domestications of *Phaseolus* beans.

In the long run, we are looking forward to seeing more coherent and systematic samplings of replicated contrasting populations across hierarchically nested levels of divergence in all kinds of species and biomes. Understanding the extent of repeatability and the causes of genomic divergence has always been challenging but the field is now moving forward toward a more cohesive framework. New ways in characterizing obscure genomic features promise aiding our understanding on how the genomic landscape of divergence is shaped.

## Data Accessibility

The sample key file, pipeline configuration file, and filtered dataset are archived at the Dryad Digital Repository under doi: 10.5061/dryad.54c5q66.

## Author Contributions

AC and MC-S conceived this study with insights from MB. AC and MC-S carried out DNA extractions to produce GBS data. AC, MC-S, and PS analyzed genotyping by sequencing data. AC wrote the manuscript with contributions from all co-authors.

## Conflict of Interest Statement

The authors declare that the research was conducted in the absence of any commercial or financial relationships that could be construed as a potential conflict of interest. The reviewer MM and handling Editor declared their shared affiliation.

## Supplementary Material

FIGURE S1 Frequency distributions of relative differentiation across hierarchically nested levels of divergence in *Phaseolus* beans. Relative differentiation is calculated as the fixation index (F_ST_). Average F_ST_ values per sliding window (window size = 1 × 10^7^ bps, step size = 500 kb) were computed as follows: **(A)** between species (*P. lunatus* vs. *P. vulgaris*), **(B)** between Andean and Mesoamerican genepools of *P. vulgaris*, **(C)** between Andean I and Mesoamerican I genepools of *P. lunatus*, **(D)** between Andean I and Mesoamerican II genepools of *P. lunatus*, **(E)** between Mesoamerican I and Mesoamerican II genepools of *P. lunatus*, **(F)** between wild and cultivated accessions of Andean *P. vulgaris*, **(G)** between wild and cultivated accessions of Mesoamerican *P. vulgaris*, **(H)** between wild and cultivated accessions of Andean I *P. lunatus*, **(I)** between wild and cultivated accessions of Mesoamerican I *P. lunatus*, and **(J)** between wild and cultivated accessions of Mesoamerican II *P. lunatus*. Dashed lines indicate thresholds for the identification of outliers at an α of 0.05.

FIGURE S2 Detail of the genomic landscape of relative differentiation in *Phaseolus* beans across hierarchically nested levels of divergence. Sliding window analyses (window size = 1 × 10^7^ bps, step size = 500 kb) for relative differentiation, as measured by the fixation index (F_ST_), were computed as follows: **(A)** between species (*P. lunatus* vs. *P. vulgaris*), **(B)** between Andean and Mesoamerican genepools of *P. vulgaris*, **(C)** between Andean I and Mesoamerican I genepools of *P. lunatus*, **(D)** between Andean I and Mesoamerican II genepools of *P. lunatus*, **(E)** between Mesoamerican I and Mesoamerican II genepools of *P. lunatus*, **(F)** between wild and cultivated accessions of Andean *P. vulgaris*, **(G)** between wild and cultivated accessions of Mesoamerican *P. vulgaris*, **(H)** between wild and cultivated accessions of Andean I *P. lunatus*, **(I)** between wild and cultivated accessions of Mesoamerican I *P. lunatus*, and **(J)** between wild and cultivated accessions of Mesoamerican II *P. lunatus*. Results of all windowed analyses are plotted against window midpoints in millions of base pairs (Mb). Black and gray colors highlight different common bean chromosomes according to Schmutz et al. (2014). Gray arrows on the vertical axes mark genome-wide averages. Gray dashed horizontal lines indicate F_ST_ thresholds for the identification of outliers at an α of 0.05 (from Supplementary Figure S1). Vertical translucent boxes feature the 1 Mb flanking region of each outlier window midpoint for between- (red boxes) and within- (gray boxes) species comparisons.

FIGURE S3 Detail of the genomic landscape of divergence in *Phaseolus* beans across hierarchical nested levels. Sliding window analyses (window size = 1 × 10^7^bps, step size = 500 kb) are shown for: **(A)** relative differentiation (from Figure 3A) computed as the fixation index (F_ST_) between species (*P. lunatus* vs. *P. vulgaris*), **(B)** delta divergence (Δ_Div_), according to Roesti et al. (2014), between genepools (results from the average F_ST_ of four within-species between-genepools, Supplementary Figures S2B–E, and three between-species within-genepool, Supplementary Figures S4A–C, comparisons), **(C)** Δ_Div_ between domestication statuses for *P. vulgaris* (results from the average F_ST_ of two within-genepool wild-cultivated comparisons, Supplementary Figures S2F,G, and two between-genepool wild-wild cultivated-cultivated comparisons, Supplementary Figures S4D,E) and **(D)** Δ_Div_ between domestication statuses for *P. lunatus* (results from the average F_ST_ of three within-genepool wild-cultivated comparisons, Supplementary Figures S2H–J, and six between-genepool wild-wild cultivated-cultivated comparisons, Supplementary Figures S4F–K). Vertical translucent boxes highlight the 1 Mb flanking region of each F_ST_-based outlier window midpoint (from Figure 3) when F_ST_ was computed as follows: (red boxes, from Figure 3A) between species (*P. lunatus* vs. *P. vulgaris*), (gray boxes, from Figure 3B) between genepools (average of four within-species between-genepools comparisons, Supplementary Figures S2B–E), (green boxes, from Figure 3C) between domestication statuses for *P. vulgaris* (average of two within-genepool wild-cultivated comparisons, Supplementary Figures S2F,G) and (blue boxes, from Figure 3D) between domestication statuses for *P. lunatus* (average of three within-genepool wild-cultivated comparisons, Supplementary Figures S2H–J). Results of all windowed analyses are plotted against window midpoints in millions of base pairs (Mb). Black and gray colors highlight different common bean chromosomes according to Schmutz et al. (2014). Gray arrows on the vertical axes mark genome-wide averages.

FIGURE S4 Genomic landscape of within-population relative differentiation in *Phaseolus* beans across hierarchically nested levels. Sliding window analyses (window size = 1 × 10^7^ bps, step size = 500 kb) for relative differentiation, as measured by the fixation index (F_ST_), were computed in three between-species within-genepool comparisons as follows: **(A)** between the Andean genepool of *P. vulgaris* and the Andean I genepool of *P. lunatus*, **(B)** between the Mesoamerican genepool of *P. vulgaris* and the Mesoamerican I genepool of *P. lunatus*, and **(C)** between the Mesoamerican genepool of *P. vulgaris* and the Mesoamerican II genepool of *P. lunatus*. F_ST_ values were computed in two between-genepool wild-wild cultivated-cultivated comparisons for common bean as follows: **(D)** between wild Andean and wild Mesoamerican accessions of *P. vulgaris* and **(E)** between cultivated Andean and cultivated Mesoamerican accessions of *P. vulgaris*. F_ST_ values were calculated in three between- genepool wild-wild comparisons for Lima bean as follows: **(F)** between wild Andean I and wild Mesoamerican I accessions of *P. lunatus*, **(G)** between wild Andean I and wild Mesoamerican II accessions of *P. lunatus*, and **(H)** between wild Mesoamerican I and wild Mesoamerican II accessions of *P. lunatus*. Finally, F_ST_ values were estimated in three between-genepool cultivated-cultivated comparisons for Lima bean as follows: **(I)** between cultivated Andean I and cultivated Mesoamerican I accessions of *P. lunatus*, **(J)** between cultivated Andean I and cultivated Mesoamerican II accessions of *P. lunatus*, and **(K)** between cultivated Mesoamerican I and cultivated Mesoamerican II accessions of *P. lunatus*. Results of all windowed analyses are plotted against window midpoints in millions of base pairs (Mb). Black and gray colors highlight different common bean chromosomes based on Schmutz et al. (2014). Gray arrows on the vertical axes mark genome-wide averages. Dashed lines mark thresholds for the identification of outliers at α of 0.05. Vertical red translucent boxes show the 1 Mb flanking region of each between-species F_ST_-based outlier window midpoint (from Figure 3A).

FIGURE S5 Correlation of overall F_ST_ estimates when using the two available versions of the common bean assembly (Schmutz et al., 2014) as reference genome (*R*^2^ = 0.948, 95% CI 0.946–0.950). Chromosomes **(A)** without and **(B)** with (Pv2, Pv9, and Pv10, the latter in red) reported inversions (Bonifácio et al., 2012) are shown.

TABLE S1 Identity of the 209 common bean and Lima bean accessions used in this study. The G identification number (from the Genetic Resources Unit at the International Center for Tropical Agriculture), the status and the genepool/race identity are shown. For Lima bean, four genepool populations, according to Chacon-Sanchez and Martinez-Castillo (2017), are indicated by different symbols. Andean genepool genotypes of *P. lunatus* are divided into subgroups AI and AII, whereas Mesoamerican genepool genotypes of *P. lunatus* are divided into subgroups MI and MII. For common bean, nine within-genepool cultivated subpopulations, according to Blair et al. (2009), and five within-genepool wild subpopulations, according to Blair et al. (2012), are indicated by different symbols. Andean genepool genotypes of *P. vulgaris* are divided into cultivated subgroups NG1 and NG2 (race Nueva Granada), cultivated subgroups P1 and P2 (race Peru) and wild subgroup K5 (Andean). Mesoamerican genepool genotypes of *P. vulgaris* are divided into cultivated subgroups D1 and D2 (Durango–Jalisco complex), cultivated subgroup G (race Guatemala), cultivated subgroups M1 and M2 (race Mesoamerica), and wild subgroups K1 (Mesoamerican), K2 (Guatemalan), K3 (Colombian) and K4 (Ecuador and north-Peru).
