# Supplementary figures and images for: Does the Genomic Landscape of Species Divergence in Phaseolus Beans Coerce Parallel Signatures of Adaptation and Domestication?
Source: Front Plant Sci. 2018 Dec 19;9:1816. doi: 10.3389/fpls.2018.01816 (PMC6306030; doi:10.3389/fpls.2018.01816)

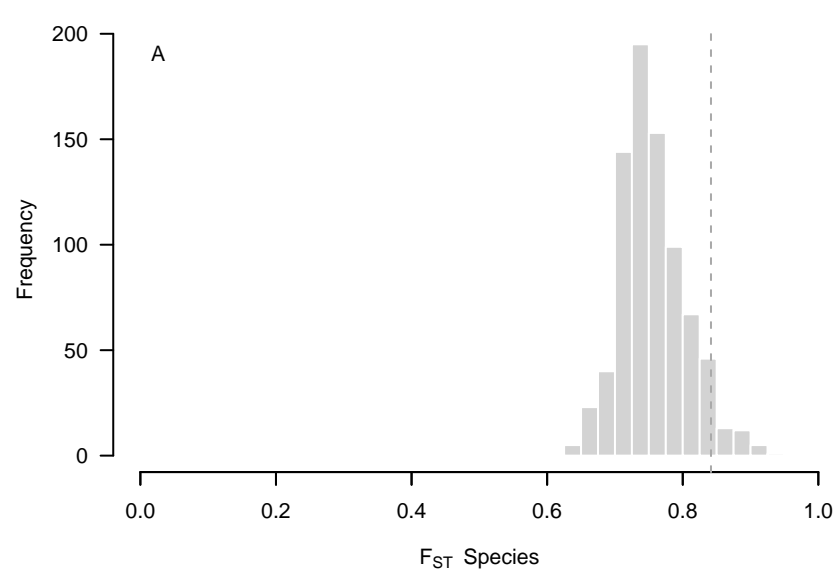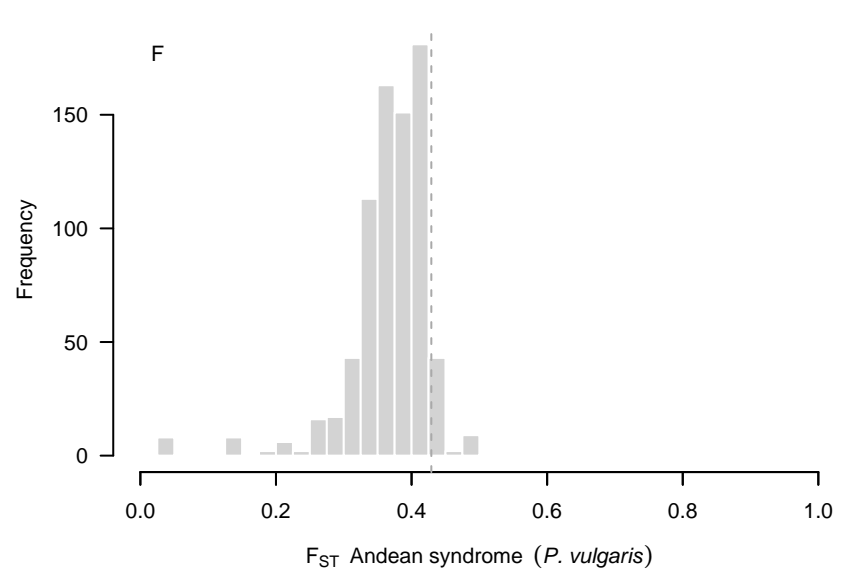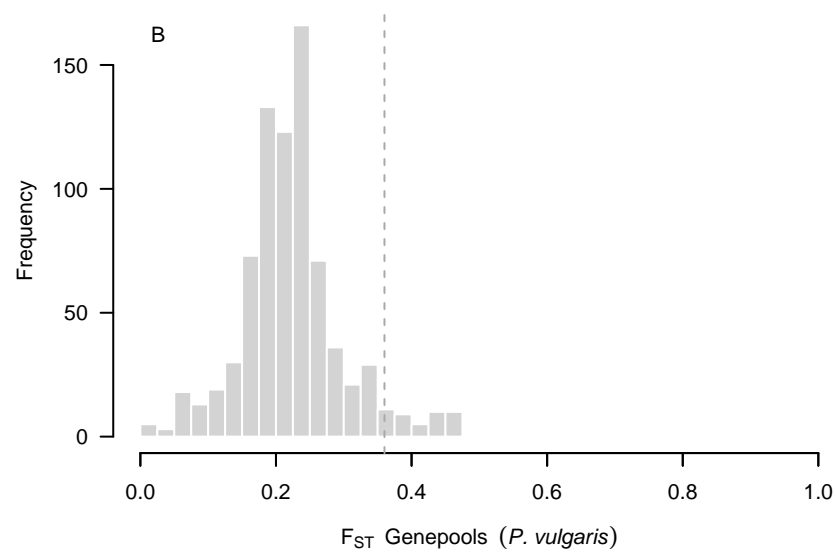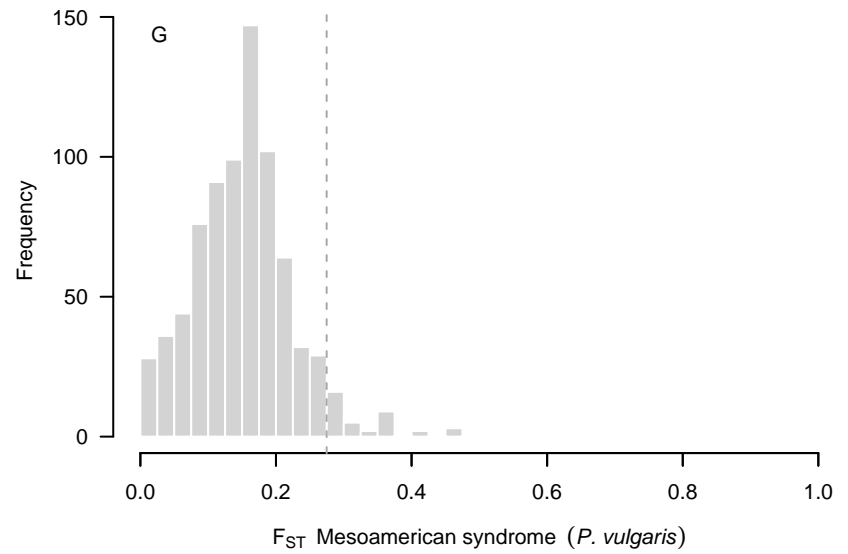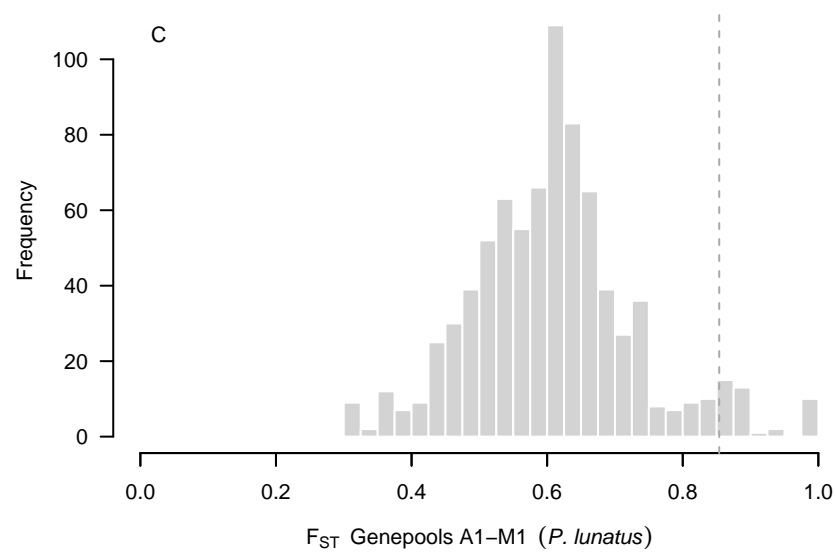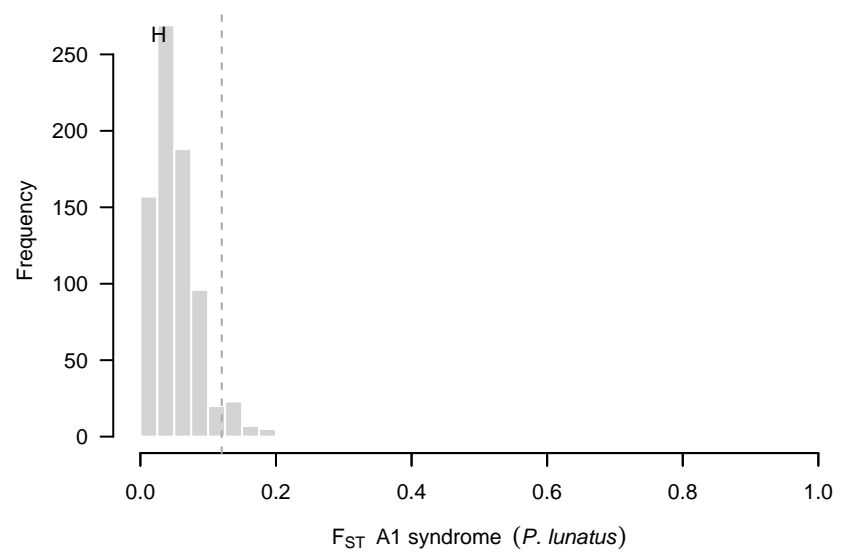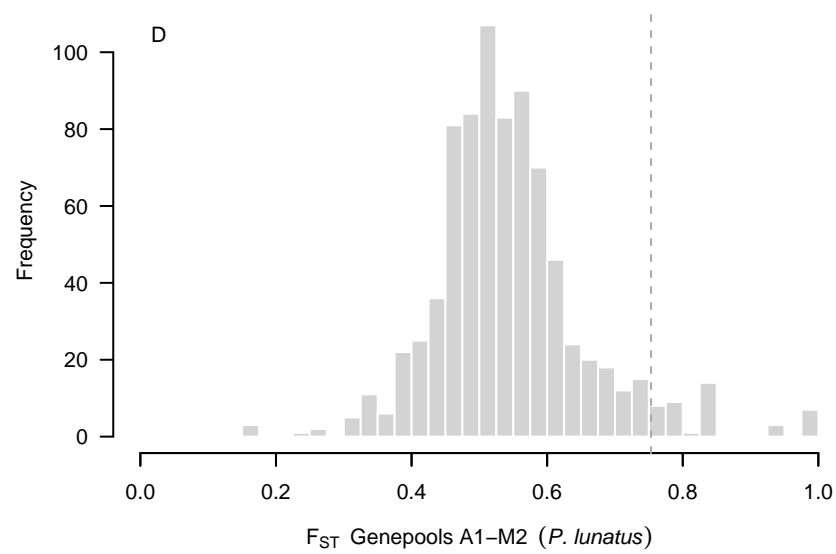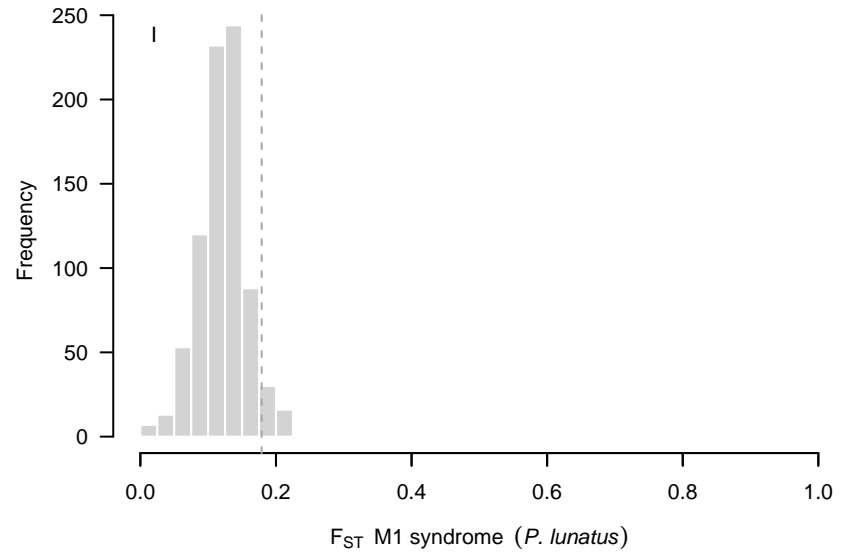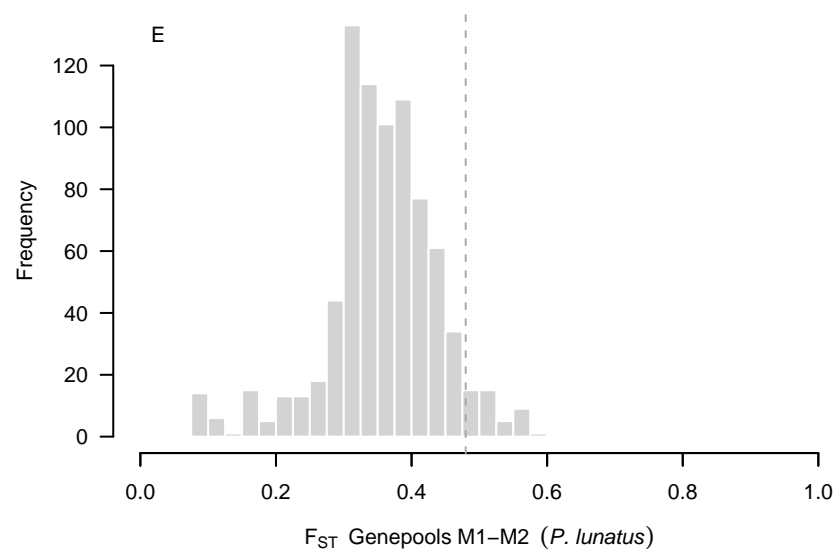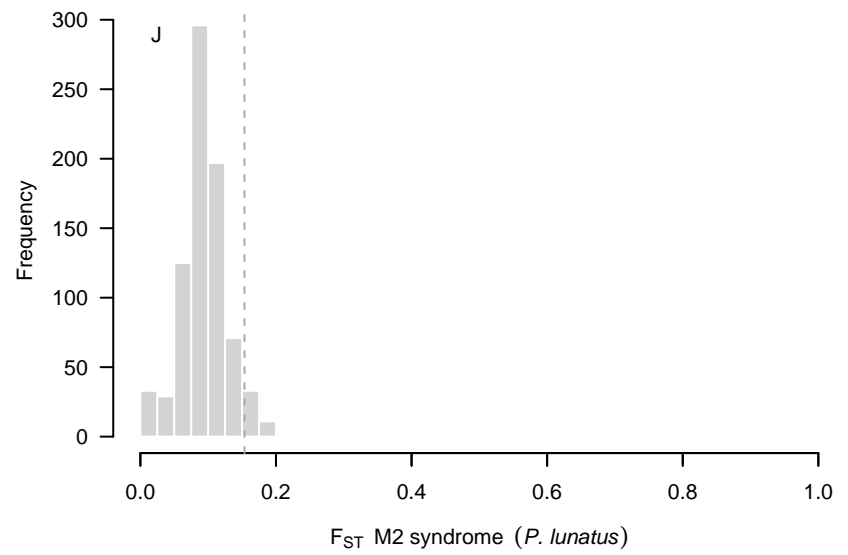

Supplement: FIGURE S1 — Frequency distributions of relative differentiation across hierarchically nested levels of divergence in Phaseolus beans. Relative differentiation is calculated as the fixation index (FST). Average FST values per sliding window (window size = 1 × 10^7^ bps, step size = 500 kb) were computed as follows: (A) between species (P. lunatus vs. P. vulgaris), (B) between Andean and Mesoamerican genepools of P. vulgaris, (C) between Andean I and Mesoamerican I genepools of P. lunatus, (D) between Andean I and Mesoamerican II genepools of P. lunatus, (E) between Mesoamerican I and Mesoamerican II genepools of P. lunatus, (F) between wild and cultivated accessions of Andean P. vulgaris, (G) between wild and cultivated accessions of Mesoamerican P. vulgaris, (H) between wild and cultivated accessions of Andean I P. lunatus, (I) between wild and cultivated accessions of Mesoamerican I P. lunatus, and (J) between wild and cultivated accessions of Mesoamerican II P. lunatus. Dashed lines indicate thresholds for the identification of outliers at an α of 0.05. [file Data_Sheet_1.PDF]

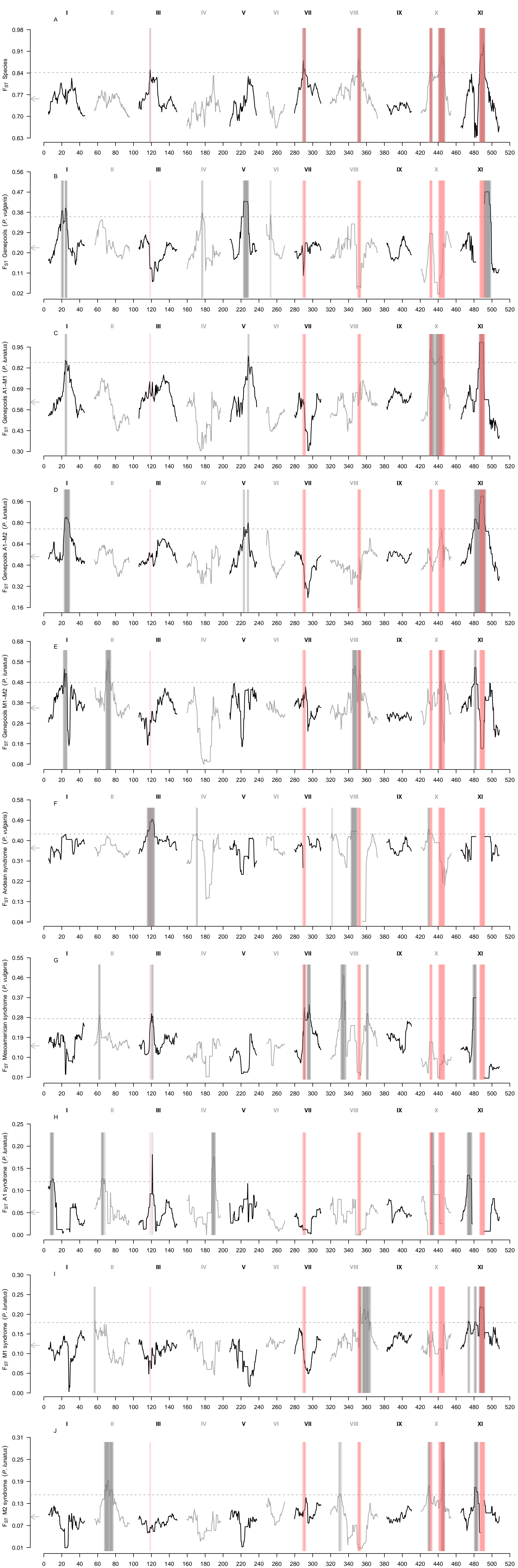

Supplement: FIGURE S2 — Detail of the genomic landscape of relative differentiation in Phaseolus beans across hierarchically nested levels of divergence. Sliding window analyses (window size = 1 × 10^7^ bps, step size = 500 kb) for relative differentiation, as measured by the fixation index (FST), were computed as follows: (A) between species (P. lunatus vs. P. vulgaris), (B) between Andean and Mesoamerican genepools of P. vulgaris, (C) between Andean I and Mesoamerican I genepools of P. lunatus, (D) between Andean I and Mesoamerican II genepools of P. lunatus, (E) between Mesoamerican I and Mesoamerican II genepools of P. lunatus, (F) between wild and cultivated accessions of Andean P. vulgaris, (G) between wild and cultivated accessions of Mesoamerican P. vulgaris, (H) between wild and cultivated accessions of Andean I P. lunatus, (I) between wild and cultivated accessions of Mesoamerican I P. lunatus, and (J) between wild and cultivated accessions of Mesoamerican II P. lunatus. Results of all windowed analyses are plotted against window midpoints in millions of base pairs (Mb). Black and gray colors highlight different common bean chromosomes according to Schmutz et al. (2014). Gray arrows on the vertical axes mark genome-wide averages. Gray dashed horizontal lines indicate FST thresholds for the identification of outliers at an α of 0.05 (from Supplementary Figure S1). Vertical translucent boxes feature the 1 Mb flanking region of each outlier window midpoint for between- (red boxes) and within- (gray boxes) species comparisons. [file Data_Sheet_2.PDF]

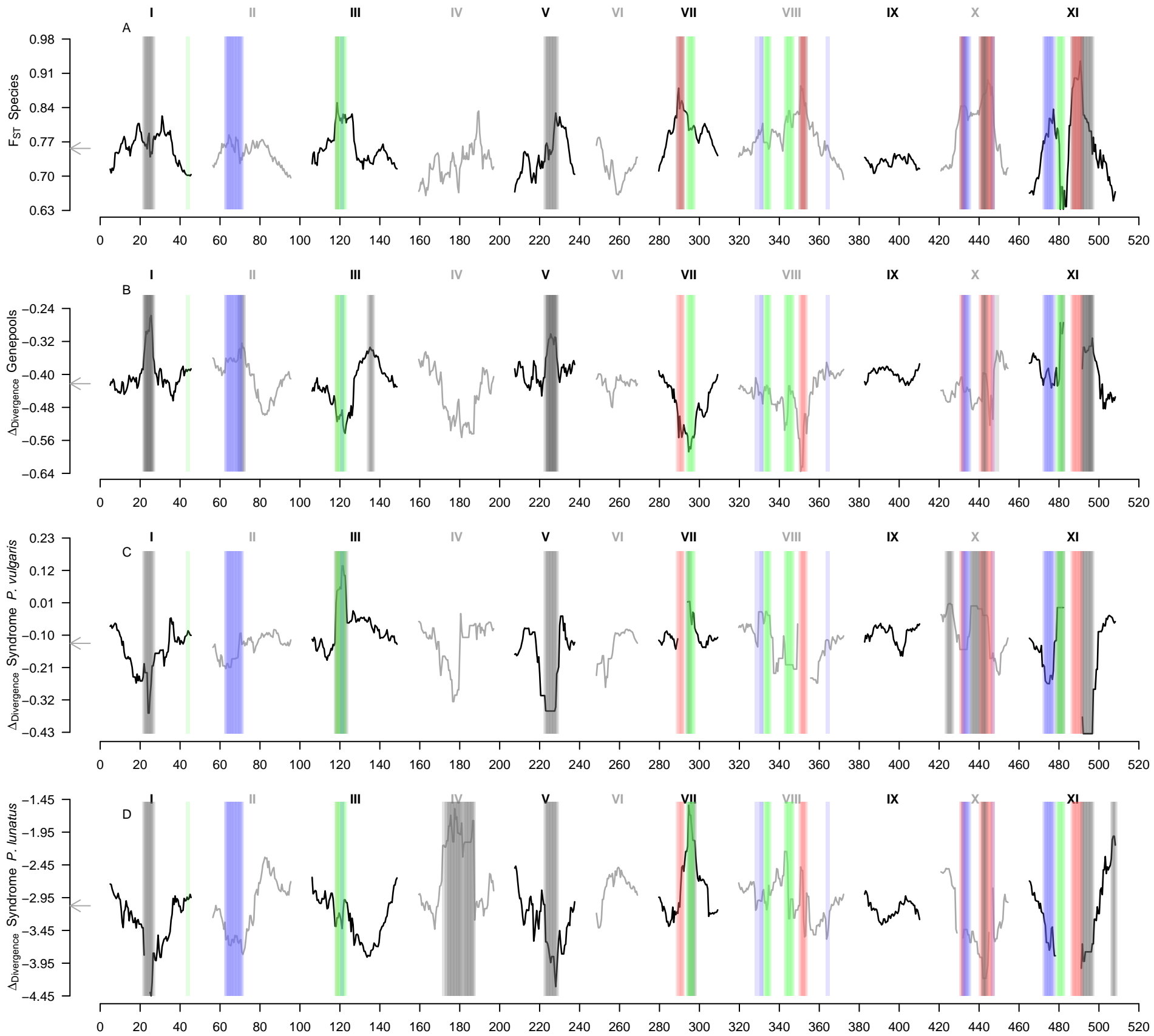

Supplement: FIGURE S3 — Detail of the genomic landscape of divergence in Phaseolus beans across hierarchical nested levels. Sliding window analyses (window size = 1 × 10^7^bps, step size = 500 kb) are shown for: (A) relative differentiation (from Figure 3A) computed as the fixation index (FST) between species (P. lunatus vs. P. vulgaris), (B) delta divergence (ΔDiv), according to Roesti et al. (2014), between genepools (results from the average FST of four within-species between-genepools, Supplementary Figures S2B–E, and three between-species within-genepool, Supplementary Figures S4A–C, comparisons), (C) ΔDiv between domestication statuses for P. vulgaris (results from the average FST of two within-genepool wild-cultivated comparisons, Supplementary Figures S2F,G, and two between-genepool wild-wild cultivated-cultivated comparisons, Supplementary Figures S4D,E) and (D) ΔDiv between domestication statuses for P. lunatus (results from the average FST of three within-genepool wild-cultivated comparisons, Supplementary Figures S2H–J, and six between-genepool wild-wild cultivated-cultivated comparisons, Supplementary Figures S4F–K). Vertical translucent boxes highlight the 1 Mb flanking region of each FST-based outlier window midpoint (from Figure 3) when FST was computed as follows: (red boxes, from Figure 3A) between species (P. lunatus vs. P. vulgaris), (gray boxes, from Figure 3B) between genepools (average of four within-species between-genepools comparisons, Supplementary Figures S2B–E), (green boxes, from Figure 3C) between domestication statuses for P. vulgaris (average of two within-genepool wild-cultivated comparisons, Supplementary Figures S2F,G) and (blue boxes, from Figure 3D) between domestication statuses for P. lunatus (average of three within-genepool wild-cultivated comparisons, Supplementary Figures S2H–J). Results of all windowed analyses are plotted against window midpoints in millions of base pairs (Mb). Black and gray colors highlight different common bean chromosomes acc [file Data_Sheet_3.PDF]

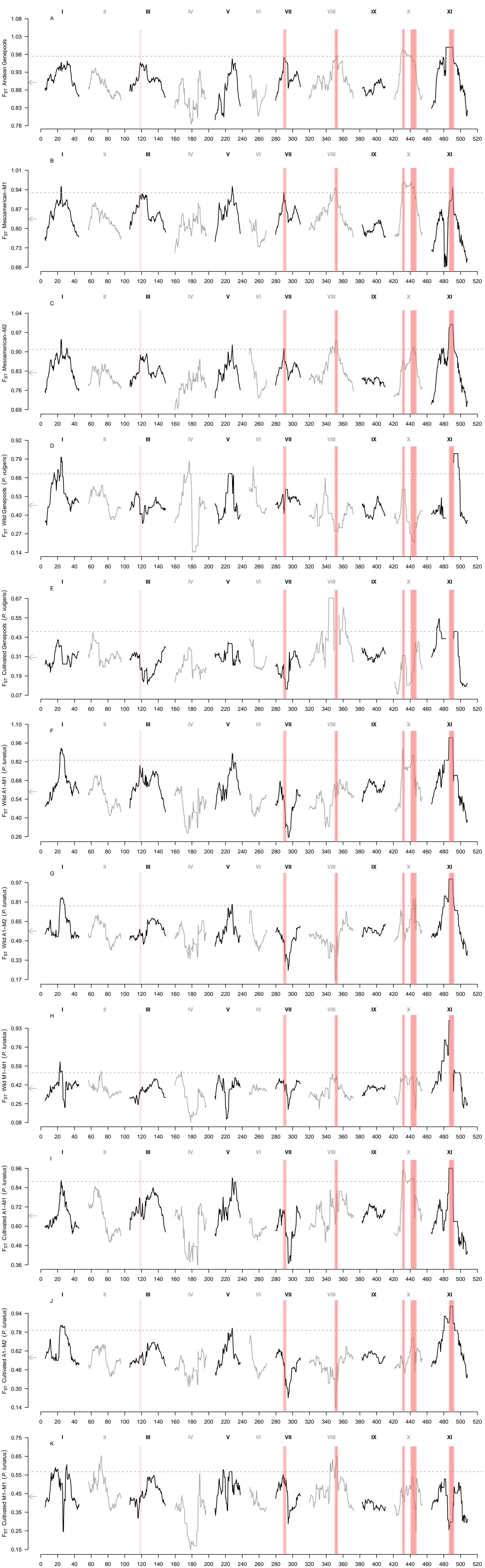

Supplement: FIGURE S4 — Genomic landscape of within-population relative differentiation in Phaseolus beans across hierarchically nested levels. Sliding window analyses (window size = 1 × 10^7^ bps, step size = 500 kb) for relative differentiation, as measured by the fixation index (FST), were computed in three between-species within-genepool comparisons as follows: (A) between the Andean genepool of P. vulgaris and the Andean I genepool of P. lunatus, (B) between the Mesoamerican genepool of P. vulgaris and the Mesoamerican I genepool of P. lunatus, and (C) between the Mesoamerican genepool of P. vulgaris and the Mesoamerican II genepool of P. lunatus. FST values were computed in two between-genepool wild-wild cultivated-cultivated comparisons for common bean as follows: (D) between wild Andean and wild Mesoamerican accessions of P. vulgaris and (E) between cultivated Andean and cultivated Mesoamerican accessions of P. vulgaris. FST values were calculated in three between- genepool wild-wild comparisons for Lima bean as follows: (F) between wild Andean I and wild Mesoamerican I accessions of P. lunatus, (G) between wild Andean I and wild Mesoamerican II accessions of P. lunatus, and (H) between wild Mesoamerican I and wild Mesoamerican II accessions of P. lunatus. Finally, FST values were estimated in three between-genepool cultivated-cultivated comparisons for Lima bean as follows: (I) between cultivated Andean I and cultivated Mesoamerican I accessions of P. lunatus, (J) between cultivated Andean I and cultivated Mesoamerican II accessions of P. lunatus, and (K) between cultivated Mesoamerican I and cultivated Mesoamerican II accessions of P. lunatus. Results of all windowed analyses are plotted against window midpoints in millions of base pairs (Mb). Black and gray colors highlight different common bean chromosomes based on Schmutz et al. (2014). Gray arrows on the vertical axes mark genome-wide averages. Dashed lines mark thresholds for the identification of outliers at α of 0.05. Vertic [file Data_Sheet_4.PDF]

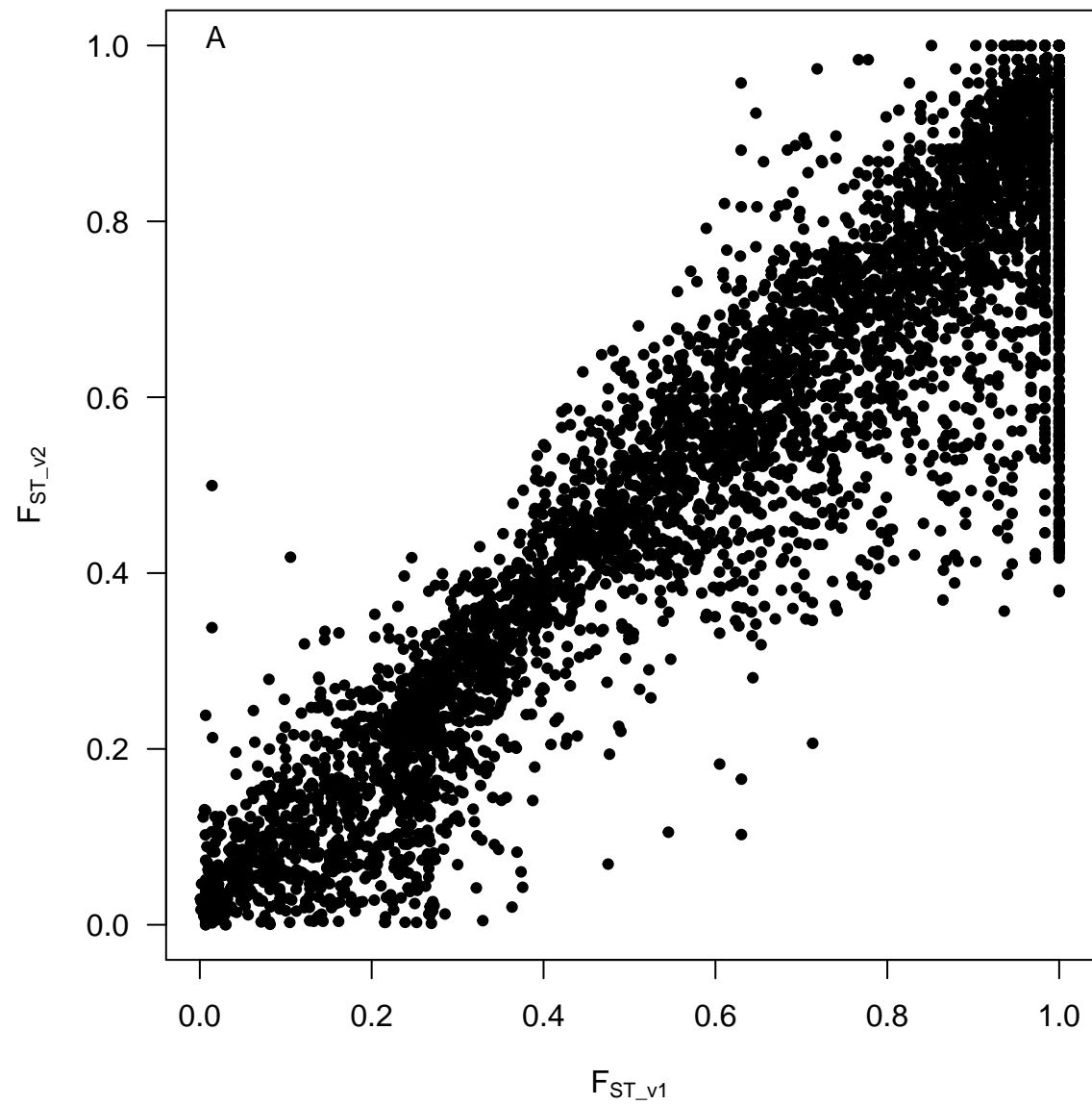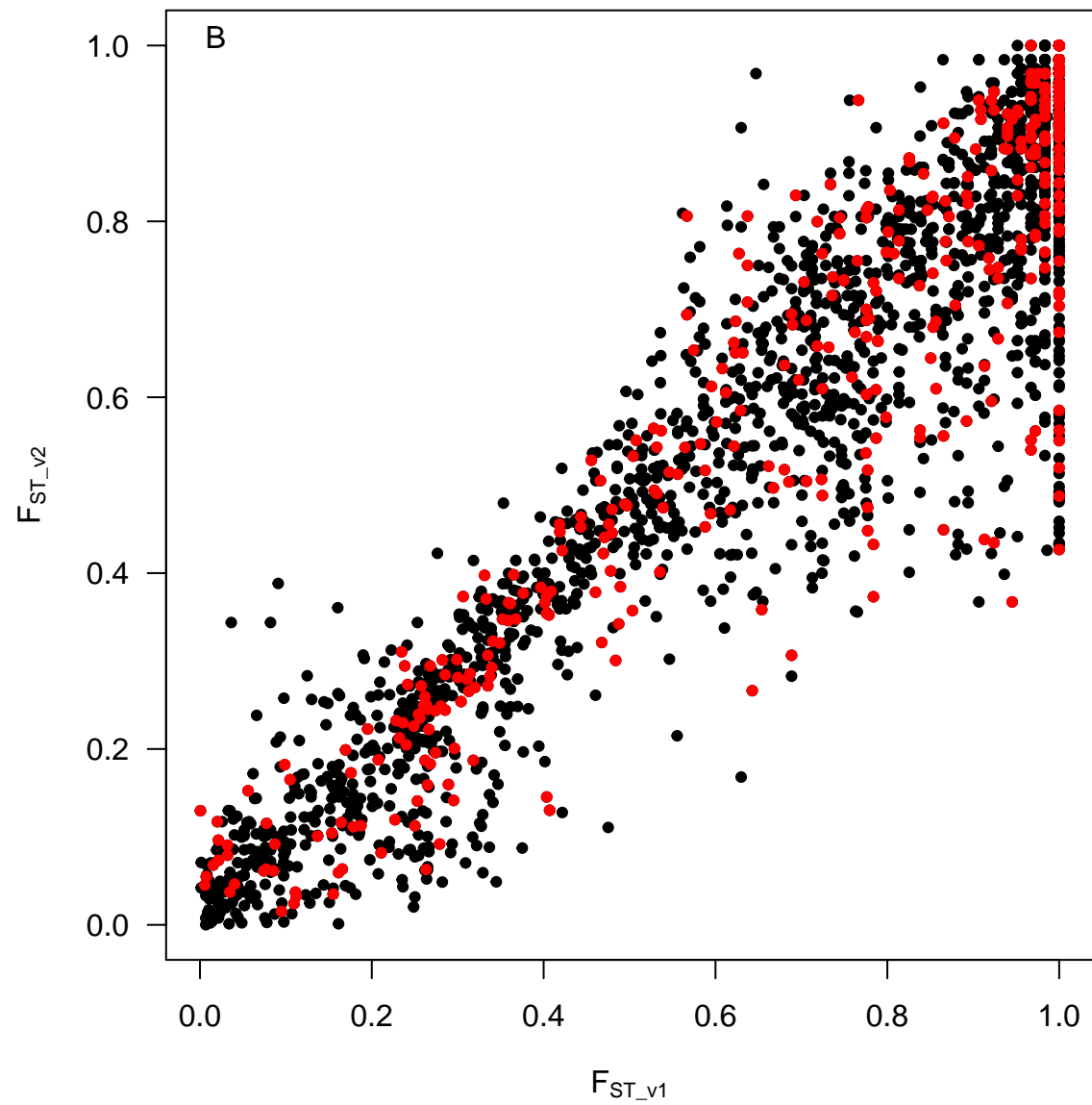

Supplement: FIGURE S5 — Correlation of overall FST estimates when using the two available versions of the common bean assembly (Schmutz et al., 2014) as reference genome (R2 = 0.948, 95% CI 0.946–0.950). Chromosomes (A) without and (B) with (Pv2, Pv9, and Pv10, the latter in red) reported inversions (Bonifácio et al., 2012) are shown. [file Data_Sheet_5.PDF]
